# Label Type Influence on DNA Translocation Velocity in Solid-State Nanopores

**DOI:** 10.1021/acsnano.6c05303

**Published:** 2026-06-17

**Authors:** Simon Brauburger, Thieme Schmidt, Filip Bošković, Ulrich F. Keyser

**Affiliations:** 2152 Cavendish Laboratory, University of Cambridge, JJ Thomson Avenue, Cambridge CB3 0US, U.K.

**Keywords:** Nanopore sensing, Solid-state nanopores, DNA nanotechnology, Molecular labeling, Polymer translocation, Translocation dynamics, Single-molecule biophysics

## Abstract

Solid-state nanopores enable single-molecule detection of long double-stranded nucleic acids and can resolve the position of site-specific molecular labels attached to a DNA carrier. These labels are employed in many applications, such as molecular barcoding, protein mapping, and structure-based DNA data storage. Often, it is implicitly assumed that these labels do not significantly perturb molecular transport through the pore. However, the magnitude of such perturbations and their potential impact on positional readout remain largely unquantified. Here, we systematically quantify how dense molecular labeling affects the translocation time of DNA carriers in solid-state nanopores using glass nanopipettes with diameters of 8–12 nm, exceeding the physical size of the labels. We employ multiple 7.2 kbp DNA carriers, each bearing up to 60 labels of a given type, including DNA nanostructures, monovalent streptavidin, and 20 kDa polyethylene glycol (PEG). Despite carrier-level differences in mass of up to 83% and charge of up to 23%, all labels produce only modest changes in global translocation time, remaining within ±15%, which is below intrameasurement variability (∼20%). This corresponds to a total velocity change of <0.25% per label. Analysis of the timing of label-associated spikes reveals that the velocity profile throughout the translocation is preserved across label types. It also indicates that 40–70% of the observed global translocation-time shift occurs while labeled regions pass through the pore, despite these regions comprising only 20% of the carrier. However, because global translocation times change only weakly and relative label positions remain largely unaffected, molecular barcoding and protein-positioning assays can generally be performed without label-specific velocity corrections under the conditions studied.

## Graphical abstract

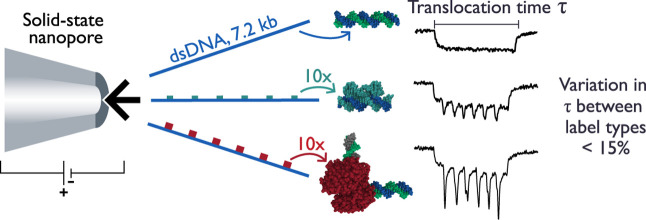

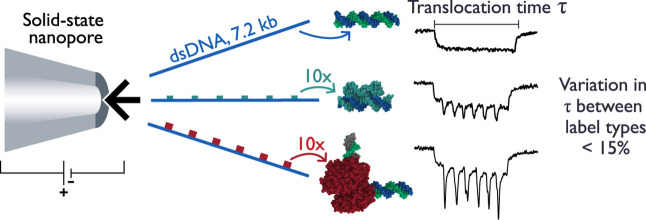

## Introduction

The analysis of small biomolecules, such as DNA, RNA and proteins is essential for deciphering fundamental biological processes, disease diagnostics and a wide range of biotechnological applications. In biosensing with solid-state nanopores (SSNPs), transient current modulations induced by sensing targets passing through a nanometer-scale pore decode rich structural information about the target.^1,2^ SSNPs are particularly well suited for the analysis of long (>1 kbp) double-stranded nucleic acids (dsNAs) in their native form and for resolving structural features that deviate from the canonical double helix.^3,4^ In addition to the characteristic constant backbone blockade of dsNA, localized structural modifications along the molecule generate secondary current spikes that report on their presence and position within the carrier.

Such structural modifications, throughout this work referred to as labels, are typically implemented using oligonucleotide-based DNA nanostructures that bind sequence-specifically to a long nucleic acid carrier. This strategy enables the programmable placement of multiple labels along a target DNA^5,6^ or RNA^7,8^ molecule. By placing different numbers of labels in different locations on multiple targets, which in turn each produce a distinct current signature with different spike depths and locations, the targets can be distinguished in a popular approach termed “barcoding”.^9^ A popular low-cost label is a double hairpin DNA nanostructure termed dumbbell.^6^ In practice, any kind of “overhanging” DNA (or RNA) sequence protruding from the carrier can function as label, as demonstrated with a range of different nanostructures.^7,10–13^ Often, multiple DNA nanostructures are necessary to obtain a resolvable current spike. To increase spike depth and therefore the signal for a smaller number of labels, the globular protein streptavidin can be bound to the DNA/RNA carrier via biotinylated oligonucleotides.^14–16^ Various other proteins have also been successfully bound to carriers and targets either directly or via a linking oligonucleotide.^4,17–19^

Apart from barcoding, labels are relevant for a number of other applications. In structure-based DNA data storage, binary information is encoded in labels attached to NA carriers.^20–22^ Utilizing modified NA carriers as molecular “fishing hooks”, the selective capture and detection of protein targets via aptamers attached to the NA^23,24^ has been shown. A similar approach, detecting genetic material of viruses via strand displacement of labels, was able to discriminate between different viral species and even variants.^25^

With the growing use of labels across SSNP applications, increasing attention has been directed toward understanding how such modifications influence translocation properties that ultimately determine resolution. Previous studies have shown that label geometry, size, and measurement bandwidth determine spike detectability and signal contrast.^6,12,26^ However, resolution in nanopore sensing depends not only on signal amplitude, but also on the translocation time τ of the molecule through the sensing region. Therefore, characterizing and controlling the average translocation velocity, defined as *v*_*t*_ = *L*_NA_/τ via the nucleic acid length *L*_NA_, has long been a central objective in nanopore research.^27–33^

However, while recent work has examined velocity effects in carriers containing dumbbells^34^ or multihelix bundles,^35^ a systematic analysis of how the type of molecular labeling alters both mean and position-resolved translocation velocity remains lacking. In particular, it is unclear whether labels can induce measurable slowdown through additional drag or modified electrophoretic forces, and whether they distort the velocity profile throughout the translocation in a manner that biases positional readout.

In this study, we explore these questions by systematically comparing the translocation velocities *v*_*t*_ of five different NA carriers to examine four commonly used label types. First, we introduce the DNA carrier design and the different label architectures (Figure 1). We then analyze changes in the average translocation velocity of the full molecule *v*_*t*_ (Figure 2). Next, position-resolved velocity changes throughout the translocation are examined (Figure 3). Finally, we discuss the implications for nanopore sensing and interpret the results in the context of the underlying transport physics.

**Figure 1:**
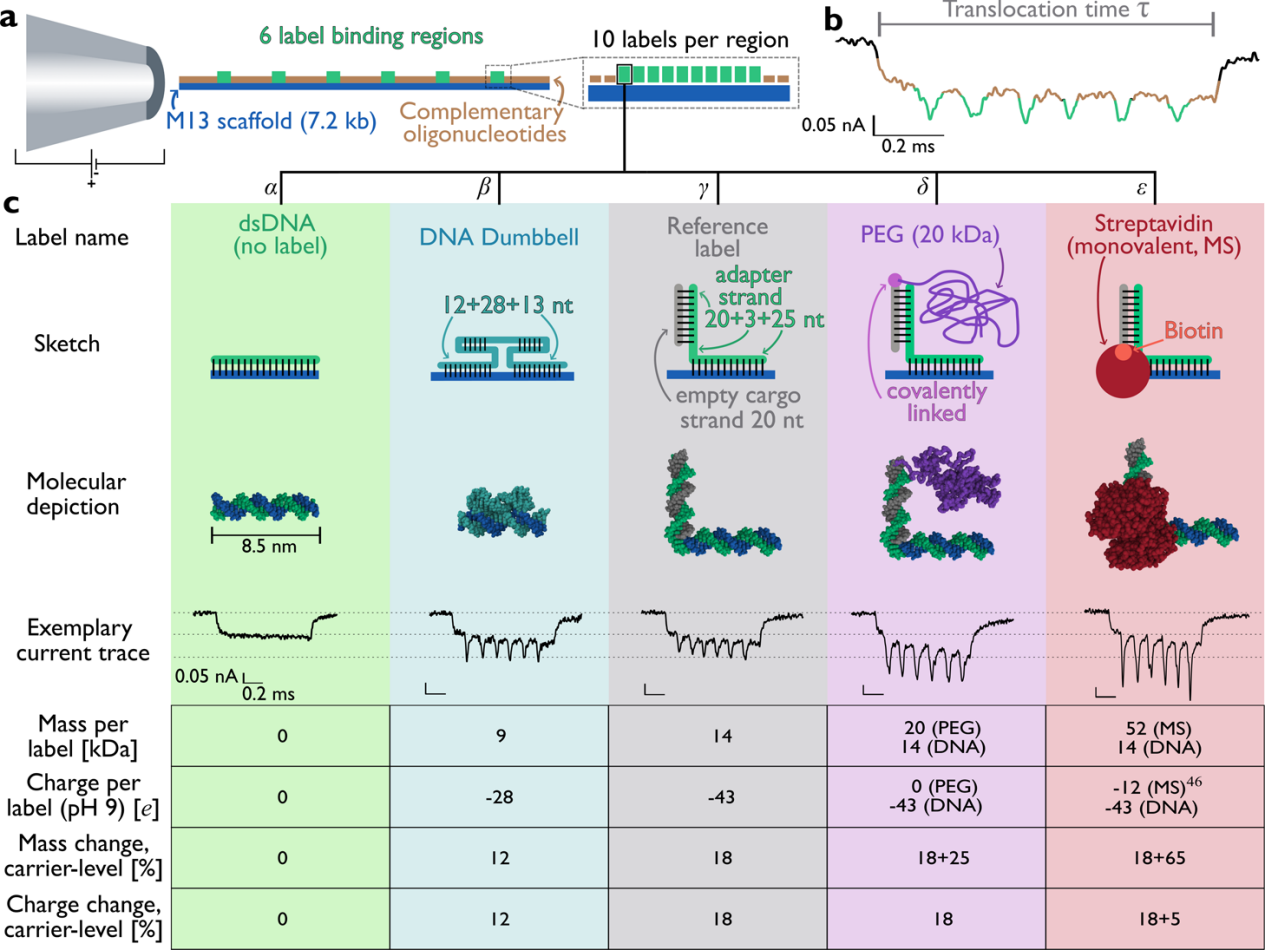
Introducing examined label types of different charge, size, weight and morphology; and experimental probe design. (a) Sketch of dsDNA carrier labeled with 60 labels, 10 per binding region, translocating into a conical nanopore. (b) Representative current trace of reference label carrier. The translocation time corresponds to the time the molecule spends in the sensing region, creating a detectable current drop (exact definition of τ see Figure S1). When passing the sensing region, the labels induce a secondary current spike (green) on top of the drop induced by the dsDNA itself (brown). (c) Overview of labels and their properties. Charges are given in terms of elementary charge *e* and for the DNA parts only include the charge of the backbone. Traces are displayed with identical time scales, current amplitudes are rescaled to demonstrate the difference in spike depth across label type. Molecular depictions created using Mol*.^36^

**Figure 2:**
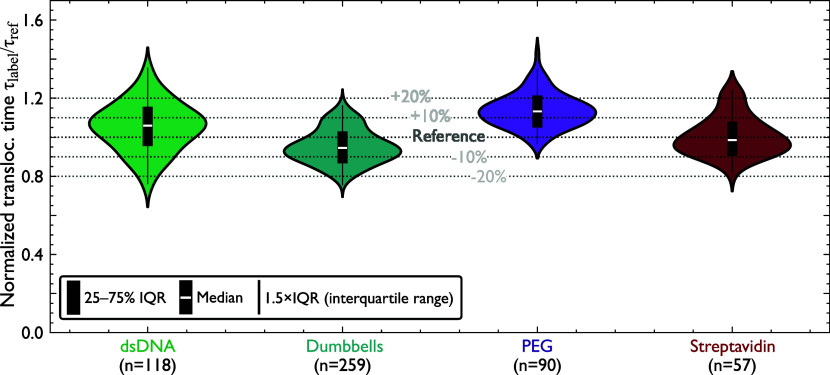
Relative changes in total translocation time τ compared to the reference label carrier. For each label, the translocation time τ_label_ was divided by τ_ref_ of the reference label carrier of the respective measurement to account for pore-to-pore variations. Changes for all label types remain within ±15%: We observe a 6.7 ± 1.2% slowdown for dsDNA without a label, a 6.0 ± 0.9% speedup for dumbbells, a 13.6 ± 2.4% slowdown for PEG, and −1.2 ± 1.9% change in translocation time for streptavidin. The reported individual values are the means, errors are the standard error of the means (SEM), not accounting for systematic errors.

**Figure 3:**
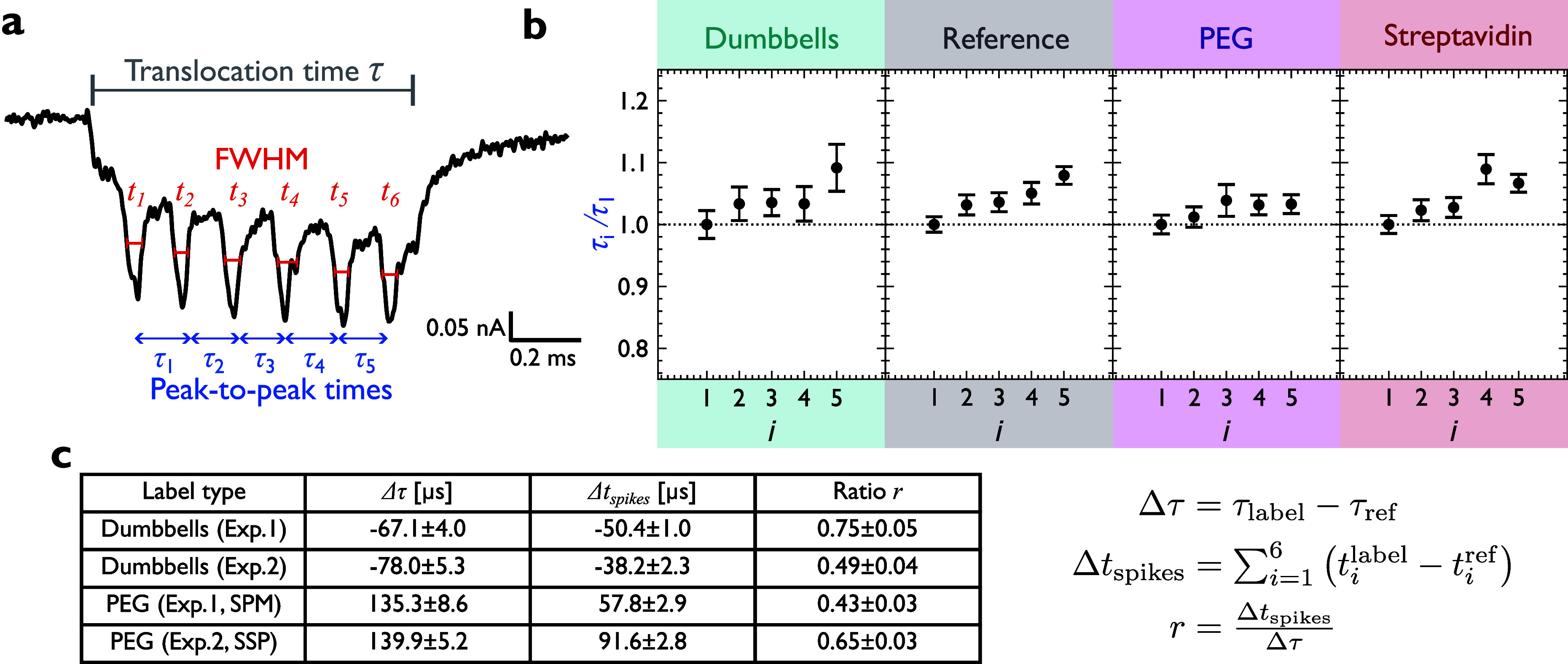
Position-resolved velocity changes during translocation (a) Definition of the temporal metrics. Spike durations *t_i_* are quantified via their full width at half-maximum (FWHM, red, full definition in Figure S9). The peak-to-peak times τ*_i_* (blue) denote the temporal spacing between consecutive spike centers and therefore correspond to the transit time of the DNA segment between two adjacent labels. (b) Normalized peak-to-peak times, indicating a slowdown throughout the first 80% of the translocation for all labels. For each label type, the segment transit times τ_i_ (i ∈ [1, ···5]) are normalized to τ_1_ of the same event. Because the interlabel distances and label regions are identical by design, deviations of τ_i_/τ_1_ from unity directly indicate relative slowing (τ_i_/τ_1_ > 1) or acceleration (τ_i_/τ_1_ < 1) of specific DNA segments during translocation. Differences between label types are lower than statistical uncertainty and remain ≲5%. (c) Comparing mean and peak-level translocation time changes from 4 different experiments with similar nanopore sizes. The cumulative change in spike durations/FWHM’s Δ*t*_spikes_ is compared to the change in total translocation time Δτ. The ratio *r* = Δ*t*_spikes_/Δτ correlates which fraction of the mean translocation time difference originates from spike broadening at the label positions versus redistribution of the interlabel segment velocities. All values significantly exceed the contour length fraction of the peak regions *L*_labels_/*L*_carrier_ = 1500 bp/7249 bp ∼ 20%. Full distributions see Figure S10. Errors in plot and table are standard errors of the mean (SEM) and do not account for systematic measurement errors. SPM and SSP definition see Methods.

## Results

### Experimental Probe Design and Label Types

In order to study the influence of the labels on *v*_*t*_, we designed a set of five DNA carrier molecules, one without labels (dsDNA) and one for each of the four examined labels. To isolate the effect of the labels, the five carriers are identical apart from the label binding regions where the different label types can bind to, as shown in Figure 1a. We used linearized M13 ssDNA as scaffold strand and covered it with complementary oligonucleotides in line with established experimental protocols,^6,14^ see Methods for details. The length of the fully assembled carrier *L*_carrier_ ∼ 7.2 kbp is representative for most NA sensing applications with SSNPs. Six label binding regions with 10 label binding sites per region were spaced equally (750 bp apart) along the M13 scaffold. The distance between regions and the number of labels for each region were chosen so the 6 current spikes induced by the 6 label binding regions can be easily distinguished at the given experimental conditions, an exemplary trace is shown in Figure 1b. The total number of labeled sites, 60, which is higher than in most applications, was chosen to enhance the magnitude of the observable effects in our nanopore diameter range (8–12 nm, diameter estimation see Table S1). All labels branch away from the main scaffold strand and electrostatic interactions of the labels with the main carrier are minimal given the high salt concentration in the measurement solution (4 M LiCl). Therefore, we expect none of the labels to change the effective contour length of the molecule, which would distort the translocation time.^33^

Figure 1c shows the different label types examined in this work. We chose a variety of more and less commonly used labels with different structure, charge and weight. The first carrier consists of the canonical dsDNA double helix structure only, without any label. The first considered label is the **dumbbell**, a compact double hairpin DNA structure that locally increases both diameter and charge. The hairpins possess some local flexibility, but their overall mobility is restricted because both ends of the ssDNA segment are anchored to the M13 scaffold, as shown in Figure 1c-β.

The second label is the **reference label**, which consists of an adapter strand hybridized to the M13 scaffold and an empty cargo strand hybridized to the adapter strand, see Figure 1(c-γ). Using this reference label allows us to isolate the effect of the attached cargo on *v*_*t*_, as the final two labels PEG and streptavidin will also be attached to a cargo strand hybridized to an adapter strand, following a strategy widely used in recent SSNP studies.^7,16,25^ Using the same cargo strand sequence for all label sites, this approach avoids the financially prohibitive alternative of synthesizing 60 individually biotinylated or PEGylated oligonucleotides. The first part of the adapter strand hybridized to the empty cargo strand is significantly shorter (∼7 nm) than the persistence length of dsNA ( $l_{p}^{\mathrm{dsDNA}}=30-60\mathrm{nm}$ depending on salt type and concentration^37^ and consequently can be considered as a “rigid rod”. The second part of the adapter strand is attached to the M13 scaffold. In between the two parts, the adapter strand contains a 3 nt single-stranded region. Considering the lower persistence length ( $l_{p}^{\mathrm{ssDNA}}∼1\mathrm{nm}$ at high ionic strengths,^38^ this label can be expected to have a certain rotational freedom induced by the ∼1 nm long single-stranded region, effectively behaving like a nunchuk with one-half fixed to the M13 scaffold.

The third label has a **20 kDa PEG molecule** bound to the cargo strand via a covalent bond. The PEG itself is assumed to contain no additional charge. While being only 50% heavier, the contour length of the PEG molecule (∼160 nm) is more than 20 times longer than that of the reference label. At the same time, its persistence length is much shorter $\left(l_{p}^{\mathrm{PEG}}∼0.4\mathrm{nm}\right)$ ,^39^ which means that it adopts a highly flexible polymer-coil with substantial conformational freedom, as depicted in Figure 1(c-δ).

The fourth label carries a tetrameric monovalent **streptavidin** molecule, shown in Figure 1(c-ε). The single active site can bind to a biotinylated nucleic acid at the 3′ end of the cargo strand. Streptavidin has a rigid globular shape and significantly higher density and mass than all of the other labels (52 kDa). Therefore, if all 60 label sites are occupied with streptavidin, the mass increase of the streptavidin carrier in comparison to the dsDNA carrier without labels is 82%; and 65% in comparison to the reference carrier. This set of four labels was deliberately chosen to encompass markedly different geometries, flexibilities, charges and masses. These labels allow us to probe how these contrasting physical features influence *v*_*t*_.

For the commonly used dumbbell and streptavidin labels, robust protocols for assembling the so-called M13 carriers are well established.^6,7,14^ For PEG, no such protocol exists. As the PEG label structure with the adapter strand and cargo strand is similar to the streptavidin label, we adapted the streptavidin assembly protocol and optimized label excess ratios using gel electrophoresis measurements (see Figure S2). Despite these optimizations, which for PEG show a clear saturation in molecular weight and therefore in the number of attached labels at higher label excess, it remains difficult to verify whether all 60 designated sites are actually occupied. However, several factors indicate a high labeling efficiency for the selected events: the sequence-specific hybridization of the adapter strands; restricting to unfolded events in the τ-based analysis approach (see Methods) as unbound oligonucleotides can induce folding;^40^ the strong affinity of the biotin–streptavidin interaction. A previous study, for example, found a binding efficiency of 87% of streptavidin to biotin-labeled carriers in solid-state nanopores.^14^ Together, these considerations suggest that the majority of labeling sites are occupied, and even if a small number of labels is vacant, we would assume this to not affect the validity of the following results.

### Mean Velocity Shifts for Different Labels

Throughout this work, the reference label and the corresponding reference carrier serve a dual purpose. Structurally, the reference label allows us to isolate the effects of PEG and streptavidin, as these molecules are attached to the carrier via the same adapter structure. Experimentally, we employ the reference carrier as an internal standard for all measurements of τ. Having a reference is motivated by pore-to-pore variations of the absolute values of τ present in SSNPs^41^ and the practical difficulty of acquiring every data set in a single pore. Therefore, we acquired data for the reference carrier in each pore alongside the label of interest. We chose the reference carrier over the dsDNA carrier as it facilitates easy detection of fragmented unfolded events via missing peaks (see Figure S3). Including fragmented dsNA in the analysis could have led to underestimations of τ. Comparing the translocation times of the reference label to those of the other labels in a given pore, we obtain relative shifts in translocation time. These are found to be consistent across multiple measurements, even though the absolute values differ, as elaborated later in the discussion. The data acquisition was performed in two different ways, single-pore multiplexing (SPM), and sequential same-pore measurements (SSP), which account for inter- and intrapore variability in different ways. They are introduced in more detail in the methods section, along with the two ways in which the data analysis was performed, τ-based and ECD-based.

Figure 2 shows the relative shifts in τ of the different label carriers with respect to the reference label carrier using results obtained with both measurement and analysis types. The distribution of the absolute τ values, including the distributions of the reference carrier for each measurement, are shown in Figure S4. For both SPM and SSP, we performed control measurements in separate pores, finding maximum deviations of <4% from the results shown here (Figures S5 and S6).

The label-induced shifts in translocation velocity are modest in magnitude, but recur consistently across control measurements and analysis methods, shown in Figures S4 and S5. For PEG-labeled carriers, a slowdown of 14–18% relative to the reference structure is observed across different experiments and analysis approaches. Likewise, dumbbell-labeled carriers show a reproducible speed-up with values ranging between 5 and 11%. In both cases, the direction and approximate magnitude of the shifts recur across data sets, indicating that these differences are intrinsic to the respective label architectures. By contrast, streptavidin-labeled carriers do not exhibit a reproducible deviation from the reference carrier. Independent measurements yield small shifts close to zero (+0.4%, −1.2%) of opposite sign.

We also performed additional controls for different operational parameters using the dumbbell and reference carrier. First, changing the voltage (300, 600, 900 mV), which did not significantly change the shifts (all showed decrease in range 6–9%, see Figure S7). Second, we tested larger pore sizes (∼14–17 nm), which showed a larger variation (decrease ranging from 3 to 14%, depending on pore, see Figure S8). Smaller pores were not tested given the more complicated translocation dynamics and increased wall interactions.^42,43^

### Position-Resolved Velocity Variation throughout Translocation

Finally, we report on how the velocity profile develops over the course of the translocation. We follow the approach of Chen et al. (2021)^34^ for measuring the ensemble average peak-to-peak times τ_*i*_. The definition of τ_*i*_ is shown in Figure 3a. As the physical peak-to-peak distance is always the same (750 bp), changes in τ_*i*_ throughout the translocation can be interpreted as velocity changes throughout the translocation. For dumbbells, reference, PEG and streptavidin, the velocity profiles are shown in Figure 3b. The data shows a small increase in τ_*i*_/τ_1_, corresponding to a small slowdown over the course of the translocation (5–10%) for all sample types. The observed differences between sample types are on the order of magnitude of the SEMs. For the dsDNA carrier, where no spikes are present, this analysis cannot be performed. Figure 3c shows the differences in translocation time between the reference and PEG and dumbbell measurements Δτ; and the differences in spike width Δ*t*_spikes_, quantified via the full width at half-maximum (FWHM) across different label types.

## Discussion

### Limited Impact of Labels on Translocation Velocity and Velocity Profile

We examined the effect of five different label types attached to a dsDNA carrier on the translocation velocity *v*_*t*_ through a solid-state glass nanopore by observing the translocation time τ. The measurements were performed in nanopipettes at high salt (4 M LiCl) for nanopore diameters around 10 nm. Those pore diameters exceed the diameter of the dsDNA (2 nm) and the dsDNA-label constructs (<7 nm). Our measurements reveal that in this diameter regime, the influence of commonly used labels on the translocation velocity is surprisingly modest. As shown in Figure 2, for a dsDNA carrier bearing up to 60 labels the observed relative shifts in τ for different labels compared to a bare dsDNA molecule all remained below 15%, corresponding to a change of ≲0.25% per label. By using the reference structure as an internal standard, the measured shifts were found to be systematic and reproducible across multiple pores and measurements.

These shifts are smaller than the intrinsic variability of τ typically observed in nanopore translocation experiments, where relative spreads of σ_τ_/τ ≳ 20% occur even under fixed experimental conditions.^42,44^ In addition, pore-to-pore differences introduce an additional level of variation of similar magnitude between measurements. Even for identical operational parameters and similar open current level (indicating similar pore size), variations in τ of around 20% are common^33,41^ and also observed in this work: For the reference structure alone, absolute translocation times ranged from τ = 900 to 1100 ms (Figures S4 and S5). Experimentally, this places label-induced shifts below both intrinsic event-to-event fluctuations and device-level variability. As a result, even though they introduce an additional source of variability, labels are neither the dominant constraint on how accurately events can be classified based on τ within a single measurement, nor on comparing τ across multiple measurements. Still, if one wishes to correct for the label-induced shifts for high-precision measurements, the corresponding observed shifts per individual label are −0.21% per dumbbell, −0.11% per reference label, +0.12% per PEG + reference and −0.13% per streptavidin + reference. Note that the individual reported shifts are with respect to the unlabeled dsDNA carrier, not the reference carrier which was used as internal standard in the measurements. These values are obtained by dividing the reported shifts by the number of labels (60), assuming a binding efficiency of 100%. The real binding efficiency might be slightly lower, despite our optimizations for the PEG carrier and the established protocols for the other carriers. The reported shifts per value therefore present a lower limit. Generally, these numbers should be seen more as indicative trends than exact values, especially when changing operational parameters such as the pore size. For smaller pores, wall-NA interactions can be expected to significantly increase.^42,43^ For larger pores (see Figure S8), we also observed larger variability in the shifts, again showing the significance of pore-to-pore variability even for similar diameters/current magnitudes. For lower and higher voltage (300–900 mV) the shifts appeared to be somewhat conserved, however this is not generalizable to other labels and other combinations of operational parameters. Especially for large numbers of labels as in applications like structure-based DNA data storage,^20,21^ more detailed characterizations of the exact shift for the application-specific conditions may be necessary.

The magnitude of the label-induced shifts is also significantly smaller than changes in τ obtainable by tuning operational parameters such as voltage or salt type and concentration.^32^ For example, the ∼15% slowdown observed for the PEG labels could also be achieved by lowering the voltage by ∼15%, which would have little effect on other relevant performance metrics. However, this comparison only applies to *global* average velocity effects affecting the entire molecule. This is distinct from velocity modulations localized to the labeled regions, which will be covered in the discussion on pore-local effects below. Our results suggest that the utility of labels for strategically slowing down targets or carriers globally is very limited. We even observe the opposite effect in some cases: for the dumbbell label, a speedup of ∼12% in comparison to dsDNA is observed. Our results are consistent with prior reports of a ∼10% speedup for a very similar carrier design using dumbbells.^34^

Beyond their limited impact on the overall translocation time, labels also appear to only have a minor effect on the evolution of the translocation velocity throughout the event: as shown in Figure 3, all carriers show *a* < 10% progressive slowdown over the first 85% of the translocation regardless of label type. These results are in line with previously reported results for dumbbells^34^ and 3-helix-bundles.^43^ The differences in this slowdown between labels, described via the normalized local velocity τ_*i*_/τ_1_, are all ≲5% and smaller than statistical error. Considering the differences in charge, mass (both see Figure 1) and persistence length $\left(l_{p}^{\mathrm{PEG}}∼0.4\mathrm{nm}<l_{p}^{\mathrm{ssDNA}}∼1\mathrm{nm}<l_{p}^{\mathrm{dsDNA}}∼30-60\mathrm{nm}\right)$ ^37–39^ of the examined label types, our findings indicate that this characteristic intramolecular slowdown is rather insensitive to label properties. This observation is consistent with a picture in which the time-dependent velocity profile is set by the evolving conformation and tension propagating through the translocating nucleic acid, rather than by the presence of attached labels. Our results are relevant for DNA carriers where the contour length (here M13 scaffold ∼ 2.5 μm) significantly exceed the label size (few nm). Previous work suggests the prevalence of tension propagation for shorter NA lengths (down to 1 kbp),^43^ for even shorter lengths the relative contribution of different label types to the friction may increase further.

The label-insensitivity of the relative velocity profile is particularly relevant for applications that rely on accurate positional information along the translocating molecule. One such application is structure-based DNA data storage, where a large number of labels are used and the relative position of the spikes encoding the bits is important.^20,21^ Knowing that the relative positions are almost unaffected by labels significantly simplifies large-scale readout necessary for any data storage solution. Another application is determining the position of NA-bound proteins with nanopores as introduced earlier, with possible potential to examine RNA-protein complexes. The fact that a label, or in this case a bound protein does not significantly affect the velocity profile is important for determining the position of the binding site. For both examples, corrections for the overall velocity profile are still required. Charron et al. (2025)^43^ provide velocity profiles for various DNA lengths, pore sizes, and voltages. For dsRNA and DNA:RNA duplexes, these profiles have not been determined yet and may vary, given differences in translocation velocity,^33^ persistence length and other physical and chemical properties of those molecules.

### Physical Origins of Label-Dependent Velocity Shifts

Even though the change in velocity is only <0.25% per label for dumbbells and PEG, we can still resolve the cumulative effect of multiple labels in our measurements, leading to the observed velocity changes with label type. To rationalize our observations, it is useful to consider a minimal description of nanopore translocation in terms of an effective force balance.^26,43^ In this picture, the instantaneous translocation velocity *v*_*t*_ is set by the balance between the electric driving force *F*_drive_ acting on the molecule within the pore and the total friction force opposing the motion,

1 $$v_{t}=\frac{F_{\mathrm{drive}}}{\gamma_{\mathrm{total}}}=\frac{F_{\mathrm{drive}}}{\gamma_{\mathrm{inside}}+\gamma_{\mathrm{outside}}}$$

γ_inside_ denotes friction contributions local to the pore region, including friction between the DNA and the surrounding fluid and DNA interactions with the pore wall. γ_outside_ captures the friction contribution of the DNA part outside the confined pore region in the cis and trans chambers. This physically reductive toy model ignores all more nuanced aspects of electrophoresis, including the local momentum transfer between the charged DNA and the counterions screening it in the nanopore. γ_inside_ and γ_outside_ should therefore be regarded as effective parameters that subsume several microscopic processes rather than as sharply separable, purely hydrodynamic drag coefficients. Nonetheless, this framework provides an accessible perspective for organizing and interpreting the experimental observations.

Within this framework, the small but systematic differences in both mean and position-resolved velocity change between label types can be rationalized qualitatively by considering their charge and morphology, which comprises contour length, persistence length and the resulting effective surface area. When compared with bare dsDNA, dumbbell labels increase the effective linear charge density λ and therefore also *F*_drive_ ∼ λ.^45^ This corresponds to an overall increase of the effective charge density $\lambda_{\mathrm{overall}}^{\mathrm{dumbbell}}\approx 1.12\lambda_{\mathrm{overall}}^{\mathrm{dsDNA}}$ on the carrier-level and a local increase of $\lambda_{\mathrm{local}}^{\mathrm{dumbbell}}\approx 1.56\lambda_{\mathrm{local}}^{\mathrm{dsDNA}}$ in the labeled regions. As they are linked with both ends to the carrier, the dumbbells remain relatively compact, and the additional friction induced by the dumbbells protruding from the carrier appears to not fully compensate the increase in *F*_drive_, leading to the observed overall speed-up of around ∼10%, in line with previously reported results.^34^ The magnitude of this change is similar to the overall increase of charge in λ_overall_ (12%). The reference label carries an even higher excess charge than dumbbells $\left(\lambda_{\mathrm{overall}}^{\mathrm{reference}}\approx 1.18\lambda_{\mathrm{overall}}^{\mathrm{dsDNA}}\right)$ . However, it translocates slower than dumbbells, indicating that the reference label architecture increases friction significantly according to eq 1. This can be attributed to its greater conformational freedom in comparison to dumbbells due to the more flexible single-stranded linker (3 nt) between the adapter and cargo strands, potentially allowing for the dissipation of energy via local torsion and increasing local friction due to higher label-fluid or label-wall interactions. Considering its shorter persistence length but longer contour length $\left(l_{c}^{\mathrm{PEG}}∼160\mathrm{nm}\gg l_{c}^{\mathrm{ref}}∼7\mathrm{nm}\right)$ , the PEG labels can be expected to form highly extended, flexible polymer coils with large effective surface area which enhance friction. At the same time, the PEG chain is not expected to introduce additional charge. Therefore, we can expect the flexibility of the PEG molecule to be responsible for the observed modest slowdown in comparison to the reference. Finally, monovalent streptavidin, for which no significant velocity change is observed, is a rigid, compact globular protein. Even though it is the heaviest label, its small hydrodynamic cross-section and limited conformational freedom appears to be compensated by its charge (−12 *e*)^46^ at the given experimental conditions (pH 9) when compared to the reference.

These observations show that the morphology of the labels can significantly change the friction the molecule experiences. Therefore, the assumption made in previous work^26^ that the total friction of the molecule is not changed by the presence of labels, i.e., $\gamma_{\mathrm{total}}^{\mathrm{labelled DNA}}=\gamma_{\mathrm{total}}^{\mathrm{dsDNA}}$ , does not hold true generally. It may match observations with a small number of dumbbell labels, where the increase in friction is outcompeted by the local charge increase and resulting increase in driving force. However, our observations indicate that this assumption does not represent the underlying physics accurately. While the contribution of the labels to the total friction is smaller than that of the dsDNA strand itself, it is not negligible, especially when shifting from e.g., the rigid dumbbells to more flexible label geometries like the reference label or PEG. In the next subsection, we will examine if this change in friction is exerted mainly by the part of the NA inside or outside the pore, described by γ_*inside*_ and γ_*outside*_, respectively.

Before moving on, we further note that the measurement pH (9) is close to the reported *pK*_*a*_ values of guanine (9.2–9.6) and thymine (9.7–10.0) for free nucleobases in aqueous solution. Under these conditions, partial deprotonation of some bases in the double-stranded DNA carrier and in the DNA-based labels may occur, potentially introducing additional negative charge. However, nucleobase *pK*_*a*_ values can shift substantially within the helical environment of duplex DNA and depend strongly on local structure, making a quantitative estimate difficult. Regardless, any additional charge from such deprotonation would be small compared to the intrinsic negative charge of the DNA phosphate backbones of both carrier and DNA labels, and is therefore unlikely to significantly affect the trends observed.

### Both Pore-Local and Polymer-Scale Effects Contribute to Friction

Now, we will examine qualitatively to which extent the label presence influences γ_inside_ and γ_outside_ by studying the spike widths, characterized by the full width at half-maximum FWHM (definition see Figure S9). While some theoretical/computational treatments exist,^47^ too little is known about how the labels generate the current spikes to assume that the FWHM (or any other peak/spike width measure) exactly correspond to the time the labeled regions stay in the pore. However, they are surely correlated and we can compare the FWHM’s of PEG and dumbbells to that of the reference label to make qualitative statements.

When comparing to the reference, if a label does not affect γ_outside_ and only changes γ_inside_ while the label is in the pore, we can assume that the observed shifts Δτ in the total translocation time are mostly distributed among the widening of the spikes and therefore in a similar change in the sum of the FWHM’s Δ*t*_spikes_ (see Figure 3 for definition of both metrics). We would therefore expect that the ratio

2 $$r=\frac{\Delta t_{\mathrm{spikes}}}{\Delta \tau }∼1$$

Conversely, if the slowdown were governed entirely by changes in dissipation outside the pore and γ_outside_, the additional delay would be distributed evenly over the full contour length of the carrier. Only the fraction of the translocation associated with the labeled region would then contribute to spike broadening, yielding an expected ratio of *r* ∼ *L*_labelregion_/*L*_scaffold_ = 1500 bp/7250 bp ≈ 0.2. The experimentally observed values of *r*, summarized in Figure 3c, lie consistently in between these two extremes, indicating that labels affect both γ_inside_ and γ_outside_. Apart from shortcomings of the FWHM extraction method, the significant variations in the r-values between experiments could be attributed to pore-to-pore variations in geometry and surface charge. The significant local speed-up of dumbbells in the nanopore is in line with previously reported results.^26^ The differences in morphology discussed in the previous subsection can therefore be expected to affect both the interaction with the solvent and wall inside the pore (γ_inside_) and the friction of the DNA parts outside the central pore region on the cis and trans side (γ_outside_).

At this point, it is important to keep in mind that we operate in a regime where the pore diameter exceeds the label size. As shown previously, when the pore size approaches the effective diameter of the labels, local friction (and therefore the ratio *r*) can be expected to further increase.^35,43^

Assuming that the widening of the FWHM is not caused by other effects, the results in this subsection suggest that while labels may not be able to meaningfully slow molecules down *globally*, they still *locally* affect velocity. Especially the PEG label could therefore be used to create a substantial local slowdown in the labeled region. Such local modulations in velocity are to the best of our knowledge not reliably accessible with any other method or change in operational parameter.

### Signal Amplitude Differences Remain Relevant for Sensing Applications

While the influence of labels on the overall translocation velocity is limited, their impact on the ionic current signal itself is more pronounced and remains relevant for practical sensing applications. Previous work has shown that the depths of the current spikes induced by labels depend sensitively on label properties such as sequence, shape, and structure.^12^ Although these signal-related differences are not the focus of the present work, the representative traces shown in Figure 1c clearly show increased spike depths for streptavidin and PEG. Explicit data for the spike depths is shown in Figure S10.

## Conclusion and Outlook

Our results show that commonly employed labels for dsDNA sensing in solid-state nanopores can be used without label-specific velocity corrections when the pore diameter exceeds the label size. Quantitatively, even dense decoration of a 7.2 kbp dsDNA with up to 60 labels changes the mean translocation time τ by less than ±15%, corresponding to a shift of <0.25% per label relative to an unlabeled dsDNA molecule. These carrier-level variations are smaller than both event-to-event and device-level variability in τ (∼20%) and therefore do not represent the resolution-limiting factor in most experimental assays. Importantly, the velocity profile throughout the translocation is conserved across label types, implying that the relative positioning of labels in the current trace is largely unaffected.

From a physics perspective, our results indicate that the tension-propagation dynamics previously reported for dsDNA carriers^34^ remain largely unaffected by the presence of attached labels. Together with the modest changes in mean velocity, this supports a picture in which the dominant friction contributions arise from the evolving conformation of the translocating polymer and the associated tension propagation, rather than from the labels themselves. Nevertheless, label charge and morphology can still measurably perturb both pore-local and polymer-scale friction: The labeled regions comprise only ∼20% of the carrier, however, the observed change in peak widths corresponds to 40–70% the magnitude of the global change in τ. This indicates that a disproportionate part of the global change occurs while the labeled regions pass through the pore.

Looking ahead, two questions appear particularly relevant. First, it remains to be determined to what extent our observations extend to other nucleic-acid systems such as dsRNA and DNA:RNA hybrids. Similar to dsDNA, they constitute important targets in SSNP sensing, but vary in persistence length, charge distribution, and translocation velocity, which could affect especially tension propagation. Second, the question of how different label properties such as charge and structure influence current spike depth and shape. While the effect of volume and flexibility has been established for DNA-based labels,^12^ a solid understanding of the physical mechanisms underlying the label-induced current spikes is still lacking. Previous work on the dsDNA current blockade imply that both volumetric ion displacement^48^ and local ion mobility changes^49^ contribute to the signal, but the importance of either for the label spikes, to the best of our knowledge, has not been explicitly examined yet.

Overall, we demonstrate that dense molecular decoration of long nucleic acid carriers is compatible with robust nanopore readout, supporting the continued development of label-based single-molecule sensing strategies.

## Methods

### DNA Scaffold Preparation

Single-stranded M13mp18 DNA (Guild BioSciences, 7249 nt) was linearized using *Eco*RI-HF and *Bam*HI-HF restriction enzymes (New England Biolabs). Briefly, 8 μL of ssM13 scaffold (100 nM) was mixed with 1 μL of each restriction-site–forming oligonucleotide (100 μ M, Integrated DNA Technologies) in 1× CutSmart buffer (NEB). The mixture was heated to 70 °C for 30 s and slowly cooled to room temperature over 40 min before addition of 1 μL *Eco*RI-HF and 1 μL *Bam*HI-HF. Digestion was performed at 37 °C for 1 h. The linearized scaffold (7228 nt) was purified using a Monarch PCR and plasmid DNA purification kit (NEB) and eluted in 10 mM Tris–HCl (pH 8.0). DNA concentration was determined spectrophotometrically (NanoDrop 2000, Thermo Fisher Scientific).

### DNA-PEG Conjugation for PEG Cargo Strand

5′DBCO–DNA was purchased from Biomers.net and dissolved in 1× TE buffer to 100 μM concentration. This was then reacted with to 20 kDa azide PEG polymer 1 mM (H_2_O) with volume 1:1 ratio. After 24 h the solution is run on a 0.8% agarose gel, where the DNA-PEG band is cut out and purified using the Freeze and Squeeze method of BioRad. Finally, the solution is buffer exchanged into deionized water and concentrated in a SpeedVac.

### Preparation of DNA Carrier

The DNA carrier was assembled by hybridizing oligonucleotides (oligos) to the linearized M13 scaffold. All oligos were ordered from Integrated DNA Technologies. The oligos binding directly to the carrier are organized in pools, the contents of each pool are listed in Supplementary Tables S2–S5. For the dsDNA carrier, pools 1 and 2 were used; For the dumbbell carrier, pools 1 and 3 were used; For the reference/PEG/streptavidin carriers, pools 1 and 4 were used. The reaction mixture (40 μL total volume) contained 20 nM of linearized M13 DNA and 60 nM of oligonucleotides (3-fold molar excess). Additionally, for the reference carrier, the mixture contained 6 μM of the empty cargo strand (sequence 5′ACCACTAATGAGTGATATCC) (5-fold molar excess × 60 binding sites); For the PEG carrier 12 μM of the PEG cargo strand with biotin (sequence 5′PEG-ACCACTAATGAGTGATATCC, the higher concentration for PEG was chosen following the test in Figure S2); For the streptavidin carrier 6 μM of the biotin cargo strand (sequence 5′ACCACTAATGAGTGATATCC-biotin). The monovalent streptavidin molecules themselves are added later right before loading the nanopore chip with the sample mixture to avoid sticking of carriers to each other during centrifugation and storage. Assembly was carried out in buffer containing 10 mM Tris–HCl (pH 8.0) and 10 mM MgCl_2_. The mixture was heated to 70 °C for 5 min and cooled linearly to 25 °C over 45 min. Excess oligonucleotides were removed by centrifugation using Amicon Ultra 0.5 mL 100 kDa filters (Millipore) and washing twice with 10 mM Tris–HCl, 0.5 mM MgCl_2_ (pH 8.0). The purified carrier was eluted by inverting the filter and spinning for 2 min at 1000 × g. The final concentration was quantified by NanoDrop spectrophotometry and stored at 4 °C. All measurements were performed within 1 week of carrier assembly, consistent with previous reports on the stability of DNA origami structures.^50^

### Nanopore Chip Assembly

Quartz glass capillaries (0.5 mm OD, 0.2 mm ID; Sutter Instruments) were pulled using a P–2000 laser puller (Sutter Instruments) with parameters similar to those described previously.^6^ The resulting nanopipettes had tip diameters of 10 ± 2 nm, calculated from the conductance model and geometry parameters described before.^14^ Nanopipettes were integrated into polydimethylsiloxane (PDMS) microfluidic chips fabricated using a 10:1 base-to-curing-agent ratio (Sylgard 184, Dow Corning). The chips were plasma-bonded (Femto, Diener Electronic) to glass slides and filled with 4 M LiCl (pH 9) before measurement.

### Nanopore Measurements and Analysis

All measurements were performed in 4 M LiCl (pH 9) using an Axopatch 200B amplifier (Molecular Devices). The pH was set to 9 to ensure a stable nanopore surface charge, which suppresses charge fluctuations that contribute to current noise,^51^ and could reduce DNA-surface interactions that can lead to transient sticking. The ionic current was low-pass filtered at 50 kHz (900CT, Frequency Devices, 8-pole Bessel) and digitized at 1 MHz using a National Instruments PCIe-6251 data acquisition card. A constant voltage of 600 mV was applied with the cis electrode held at ground potential.

Measurement type 1: In single-pore multiplexing (SPM), the reference and the label-of-interest carrier are measured in the same pore at the same time. As visible in Figure 1(c-δ, c-ε), the spikes induced by streptavidin and PEG are significantly larger in comparison to the reference label, which allows us to easily tell the two label types apart even when they are measured in the same pore at the same time. Therefore, both streptavidin and PEG are suitable for SPM. SPM is the experiment type with the highest accuracy. Apart from accounting for pore-to-pore variation, it also accounts for time-dependent biases. As they are measured simultaneously, we can expect both sample types to be affected equally by such biases and therefore to not distort the results. Examples of time-dependent biases include evaporation resulting in salt concentration increase, and baseline drift-changes in the open current level *I*_0_ over time. All these biases affect τ.^52^

Analysis type 1: τ-based analysis. To accurately estimate τ for SPM for both populations, the events need to be classified into reference/nonreference events, but also into unfolded/folded events. We directly read off τ as shown in Figure 1b using only unfolded events. The events were preclassified via various event parameters, such as electric charge deficit (ECD), mean event current $\overset{―}{\Delta I}$ and peak event current Δ*I*_max_ (see Figure S1). The assigned categories (unfolded, folded, full-length, fragmented etc.) were then carefully checked and if necessary reassigned manually. To avoid distortions, strict criteria were employed on what counts as an acceptable unfolded event, examples are shown in Figure S11. Even small folds corresponding to spikes directly at the start/end of the event were excluded. The same applies to partially fragmented carriers, which arise as a normal byproduct in the assembly processes (Figure S3), identifiable via events with less than 6 spikes.

Due to the experimental design, translocation events of the dumbbell and the reference target are basically indistinguishable due to their similar spike depth, as visible in Figure 1(c-β, c-γ). While the dsDNA carrier can be distinguished from the reference carrier, due to the lack of spikes it is impossible to tell if an event is fragmented or not, which would skew the analysis. This makes the SPM unsuitable for determining τ for dumbbells and dsDNA. However, both problems can be accounted for with a different measurement and analysis approach.

Measurement type 2: sequential same-pore (SSP) measurements. Here, the reference and the label-of-interest carrier are measured in the same pore one after the other. After the first measurement, the sample solution is flushed and filled with the second sample. As shown previously, the number of events from residual sample can be expected to be <0.5% and therefore negligible.^53^ Similar to SPM, SSP accounts for pore-to-pore variation. However, for SSP, the sequential nature of measurements makes it more vulnerable to time-dependent biases mentioned above. We tried to account for evaporation by covering the electrode access holes of the nanopore chip with multiple layers of translucent adhesive tape as increasing salt concentration over time would slow the translocation down.^32^ Monitoring the average τ over time (see Figure S1g+h) showed that this was not the case. In some experiments, where we did not seal the chip properly, a significant increase in τ over time was observable (those were not used for this study). The fact that τ remained somewhat constant over time in the experiments used for this study indicates that the change in baseline current *I*_0_ over time (see *I*_*start*_ and *I*_*stop*_ in Table S1), also appears to not significantly distort results. The quartz nanopores used in this study are very robust in shape and the geometry of the pore itself can be expected to not significantly change even over multiple experiments.^53^ We ran a control experiment with PEG using SSP, the obtained values for τ varied by 2% from the SPM result (Figure S6).

Analysis type 2: ECD-based analysis. Measuring one label type at a time also allows for another mode of validating our claims. Earlier, we rejected all partially and fully folded events. However, as shown in other works,^54,55^ the total electric charge deficit (ECD), is a conserved quantity for translocations of different configurations including folded events. All-events-histograms for the ECD generally show a clear peak, corresponding to the full-length carrier, and a tail of shorter events, corresponding to fragmented or partially assembled carriers (Figure S3). Because the ECD effectively constitutes the integral of the current trace, it allows for a good estimate of the translocation time $\tau =\frac{ECD}{\overset{―}{\Delta I}}$ via the mean current drop $\overset{―}{\Delta I}$ for unfolded events for a given translocation type, see Figure S1. As no events are discarded, a much larger amount of sample points per measurement time is possible, allowing for statistically more significant statements even for the shorter measurement times necessary for the sequential measurements to avoid baseline shifts. Comparing both unfolded-event and ECD-based analysis methods shows variations of around 2% (Figure S5), significantly smaller than most of the observed shifts shown above.

The ECD-based analysis approach also resolves the second problem noted above: while fragmentation of dsDNA carriers is not readily identifiable in the ionic-current trace, the full-length events give rise to a well-defined ECD peak. This peak allows for a reliable extraction of τ for dsDNA carriers. However, the ECD-based analysis strategy is restricted to SSP measurements, as in SPM the ECD peaks of the two species overlap strongly.

Translocation events were recorded and analyzed using custom LabVIEW and Python software.

## Supplementary Material



## Notes

Definition/visualization of various event metrics; PEG carrier protocol optimization; fragment analysis; extended data sets absolute translocation times; additional control experiments different voltages and bigger pore diameters; exemplary current traces and selection criteria; nanopore diameters; oligonucleotide sequences used for hybridization (PDF)
