# Supplementary material for: Label Type Influence on DNA Translocation Velocity in Solid-State Nanopores
Source: ACS Nano. 2026 Jun 17;20(30):21234–44. doi: 10.1021/acsnano.6c05303 (PMC13450422; doi:10.1021/acsnano.6c05303)
Supplement: Supplementary file 1 [file nn6c05303_si_001.pdf]

Supporting information

Label type influence on DNA translocation velocity in solid-state nanopores

Simon Brauburger, Thieme Schmidt, Filip Bošković, and Ulrich F. Keyser*

Cavendish Laboratory, University of Cambridge, JJ Thomson Avenue, Cambridge CB3 0US, United Kingdom

E-mail: ufk20@cam.ac.uk

Contents

SI-1	Event metrics and folding	1
SI-2	PEG assembly	2
SI-3	Fragments and degradation	3
SI-4	Extended dataset for figure 2	4
SI-5	Control 1: Additional experiments and comparisons of analysis methods	5
SI-6	Control 2: Comparison single-pore multiplexing vs. sequential single-pore measurement	6
SI-7	Control 3: Voltage dependency	7
SI-8	Control 4: Bigger pore diameters	8
SI-9	FWHM definition	9
SI-10	Current spike properties	10
SI-11	Selection examples	11
SI-12	Nanopore diameters (Table S1)	12
SI-13	Oligonucleotides used for hybridization (Tables S2 to S5)	13

SI-1 Event metrics and folding

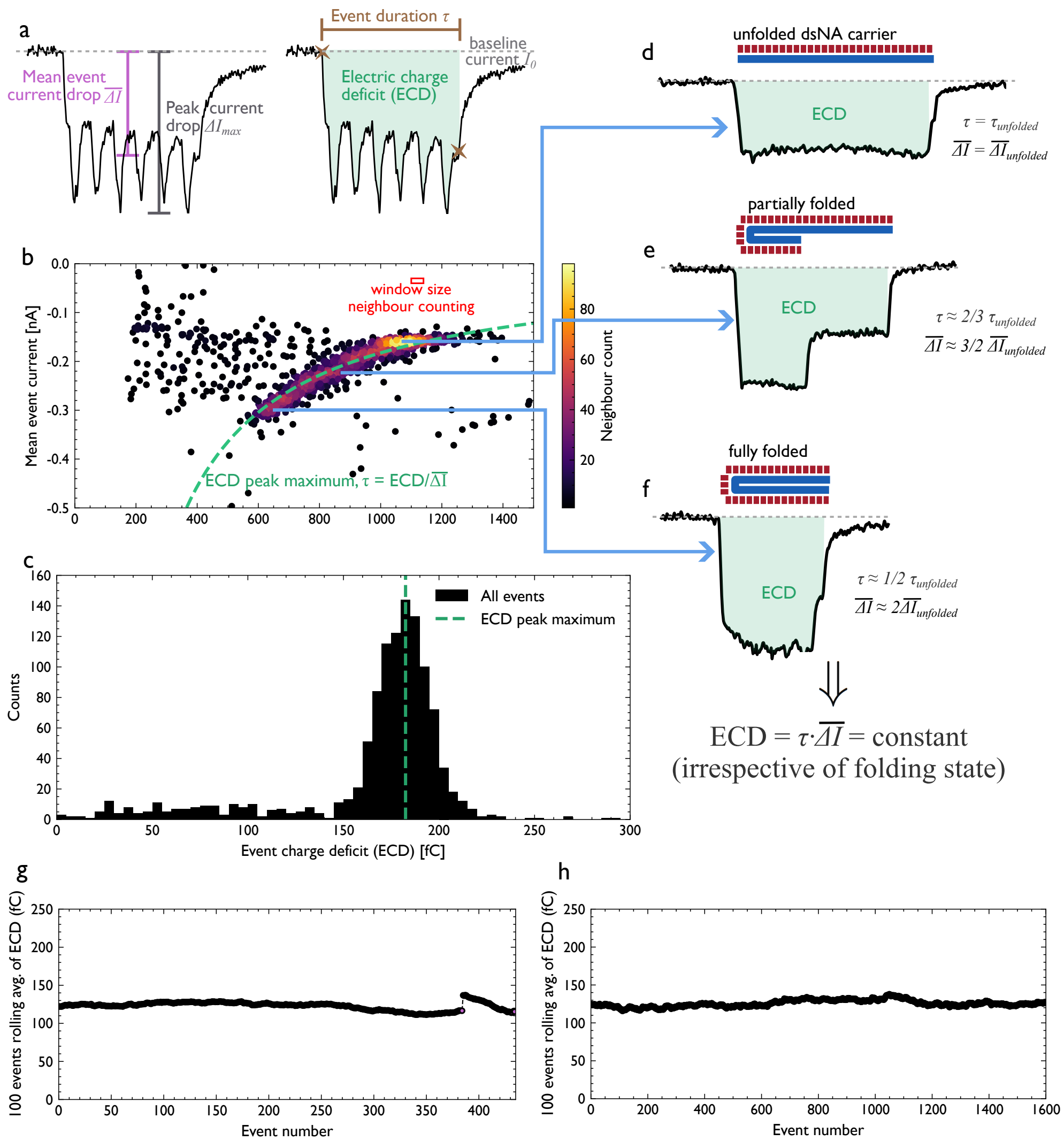

Figure S1: (a) Common metrics to analyze and pre-classify events. (b) A scatter density plot of the mean event current drop $\overline{\Delta I}$ over the translocation time τ for each event yields a typical "boomerang"-shaped curve. This curve, highlighted in green here, corresponds to a constant electric charge deficit ECD value. (c) The points around this curve correspond to a distinct peak in the ECD histogram (green curve corresponds to the same value as in b). (d-f) For unfolded, partially folded and fully folded events, the ECD constitutes a roughly preserved quantity. While τ decreases for folded events, $\overline{\Delta I}$ scales inversely proportional to it, and the ECD, its product, remains constant. Fitting the ECD peak in the histogram therefore allows us to infer the τ of unfolded events by considering the $\overline{\Delta I}$ of the unfolded events. (g+h) Rolling average (100 events) of τ over time for two exemplary measurements. Obtained via analysis method 2 (ECD-based, see methods). Minor fluctuations are observable, which can be attributed to populations of fragements translocating (see Fig. S3), or outlier events like stuck molecules which produce extremely long events (see bump around event number 380 in subfigure g). However, even over long measurements, the average τ does barely change, indicating that changes in the open current level I_0 over time (see Table S1) do not strongly affect τ.

SI-2 PEG assembly

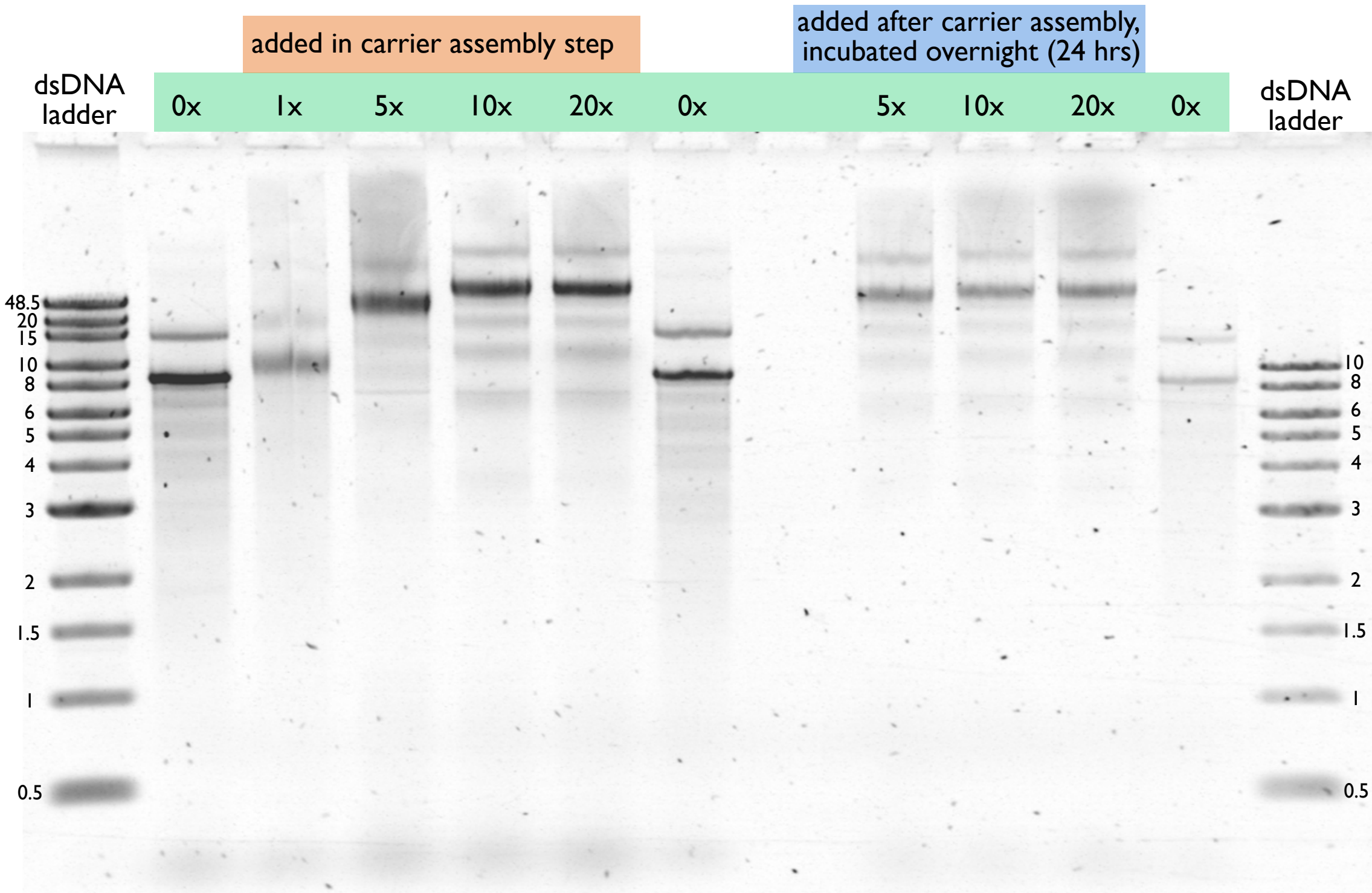

Figure S2: Test of different excess ratios of the PEG cargo strand (1x - 20x, green) and in the assembly to ensure full coverage of the label binding sites with PEG molecules. Without the PEG strand (0x), the main band of the sample is in between the 8 and 10 kbp bands of the dsDNA ladder. This is to be expected considering the $\sim$ 7.2 kbp of the complemented M13 and the additional $\sim$ 1380 nt ssDNA from the part of the adapter strand emanating from the carrier. The band at $\sim$ 15 kbp probably contains carrier duplexes. For both examined methods, adding the PEG cargo strand in the assembly (orange) and overnight after the assembly (blue), the mobility of the carrier molecule decreases significantly with higher excess. However, at 10x excess a plateau is observed and the mobility does not decrease further when increasing to 20x excess for both methods. Considering the significant mobility shift, we interpret this plateau that at 10x excess, all or most of the binding sites have a PEG molecule bound to them, and increasing the excess further does not lead to better binding. We therefore use a 10x excess in the assembly protocol. As there is only minimal difference between the direct adding and the overnight, we use the method of directly adding the PEG strand in the carrier assembly step to save time. To determine the concentration of the PEG cargo strand, we used a NanoDrop 2000 Spectrophotometer with the standard dsDNA setting. We did not know how the PEG affected the concentration measurements, another reason why we performed this test to ensure successful assembly.

SI-3 Fragments and degradation

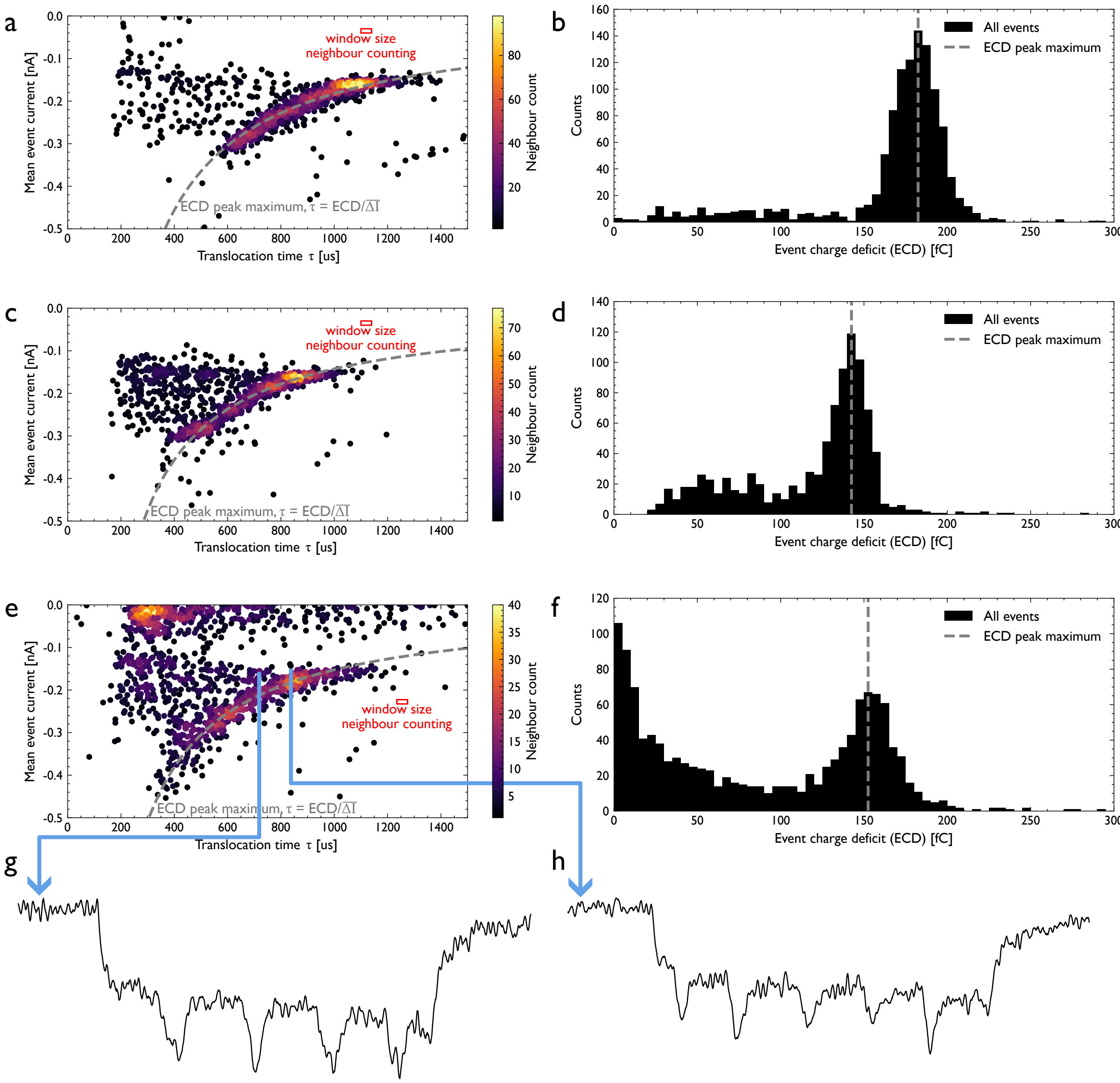

Figure S3: 3 different measurements with varying levels of degradation. (a+b) Measurement 1, little degradation, showing few scatter points left of the main boomerang in a and a clean peak in the ECD histogram in b. (c+d) Measurement 2, minor degradation. (e+f) Measurement 3, major degradation, many short fragments. (g) Fragmented molecule with only 4 out of the 6 spikes present. (h) Fragmented molecule with only 5 out of the 6 spikes present.

SI-4 Extended dataset for figure 2

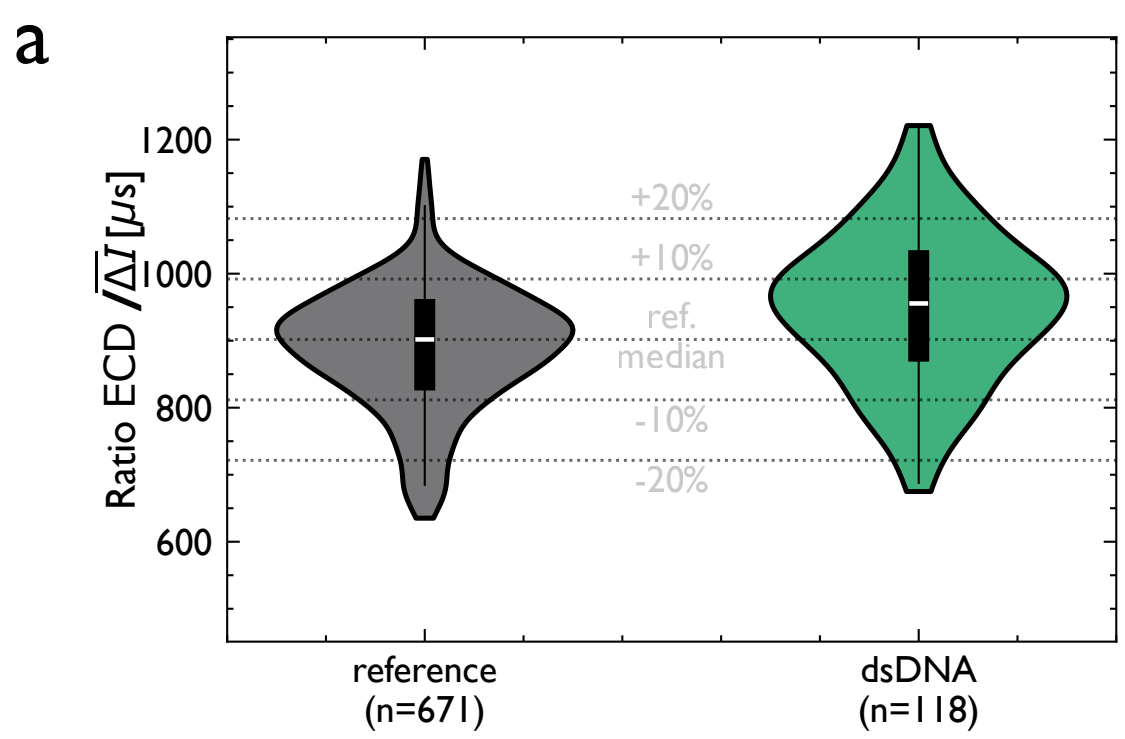

Shift: +6.70 % ± 1.23 %

Mean	889.23	949.64
SEM	3.502	10.280
Std Dev	90.71	111.67
Median	901.85	955.54
IQR	105.23	135.04

(ECD-based, SSP)

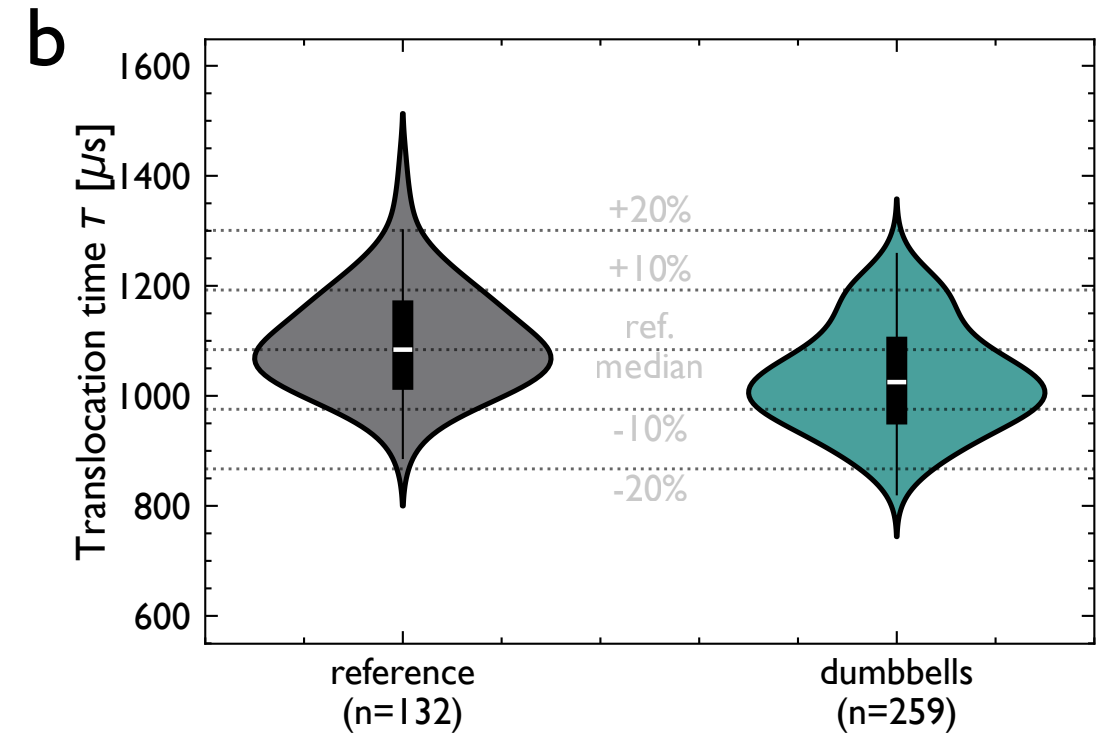

Shift: -6.03 % ± 0.87 %

Mean	1098.75	1032.44
SEM	8.057	5.811
Std Dev	92.56	93.52
Median	1084.00	1025.00
IQR	124.50	121.50

(τ-based, SSP)

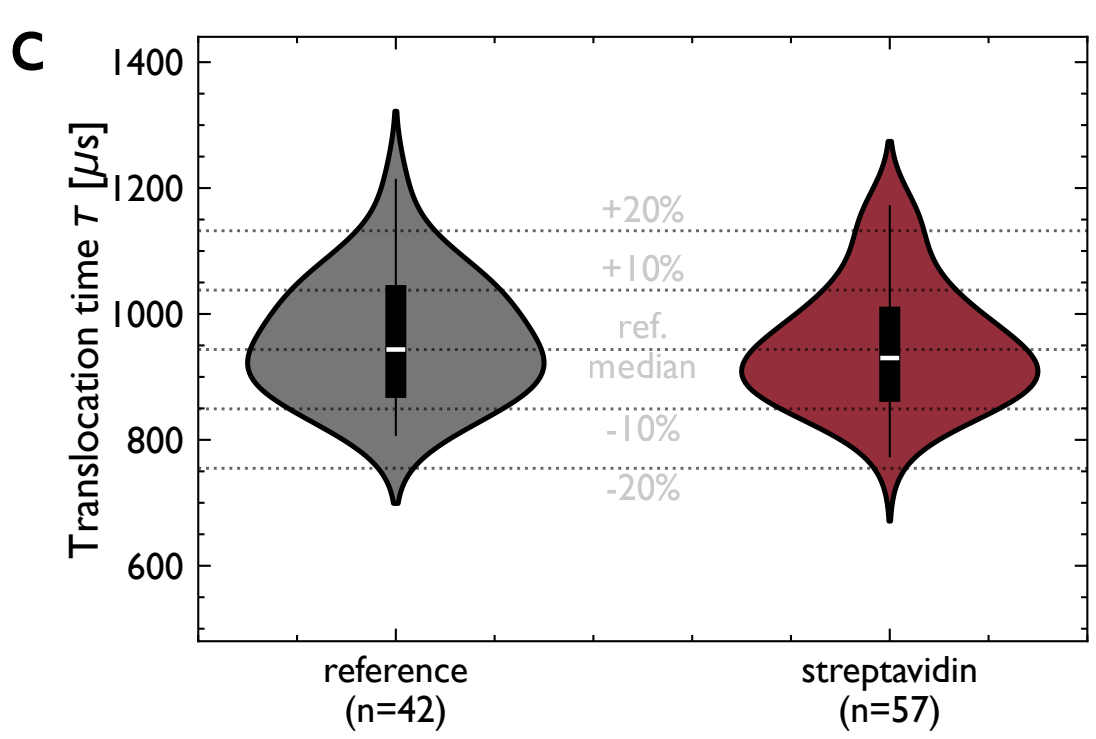

Shift :-1.17 % ± 1.94 %

Mean	960.17	948.95
SEM	14.202	12.259
Std Dev	92.04	92.55
Median	943.50	930.00
IQR	146.00	118.00

(τ-based, SPM)

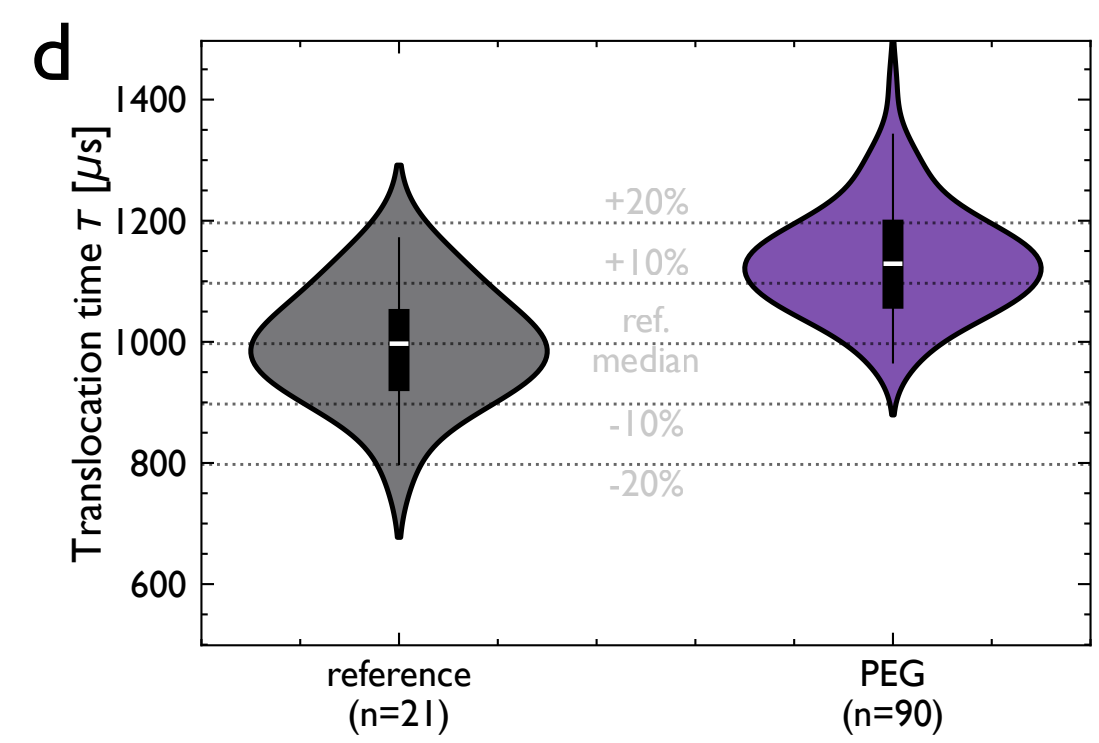

Shift: +13.56 % ± 2.38 %

Mean	997.95	1133.32
SEM	19.385	8.992
Std Dev	88.83	85.30
Median	997.00	1129.00
IQR	101.00	112.50

(τ-based, SPM)

Figure S4: Absolute τ-values of data used for Figure 2, including statistical quantities and the reference data used for normalization. (a) dsDNA, (b) dumbbells, (c) streptavidin, (d) PEG. Relative shifts calculated via $(\tau_{\text{label}} - \tau_{\text{reference}})/\tau_{\text{reference}}$ using mean values, errors propagated from standard errors of the means (SEM).

SI-5 Control 1: Additional experiments and comparisons of analysis methods

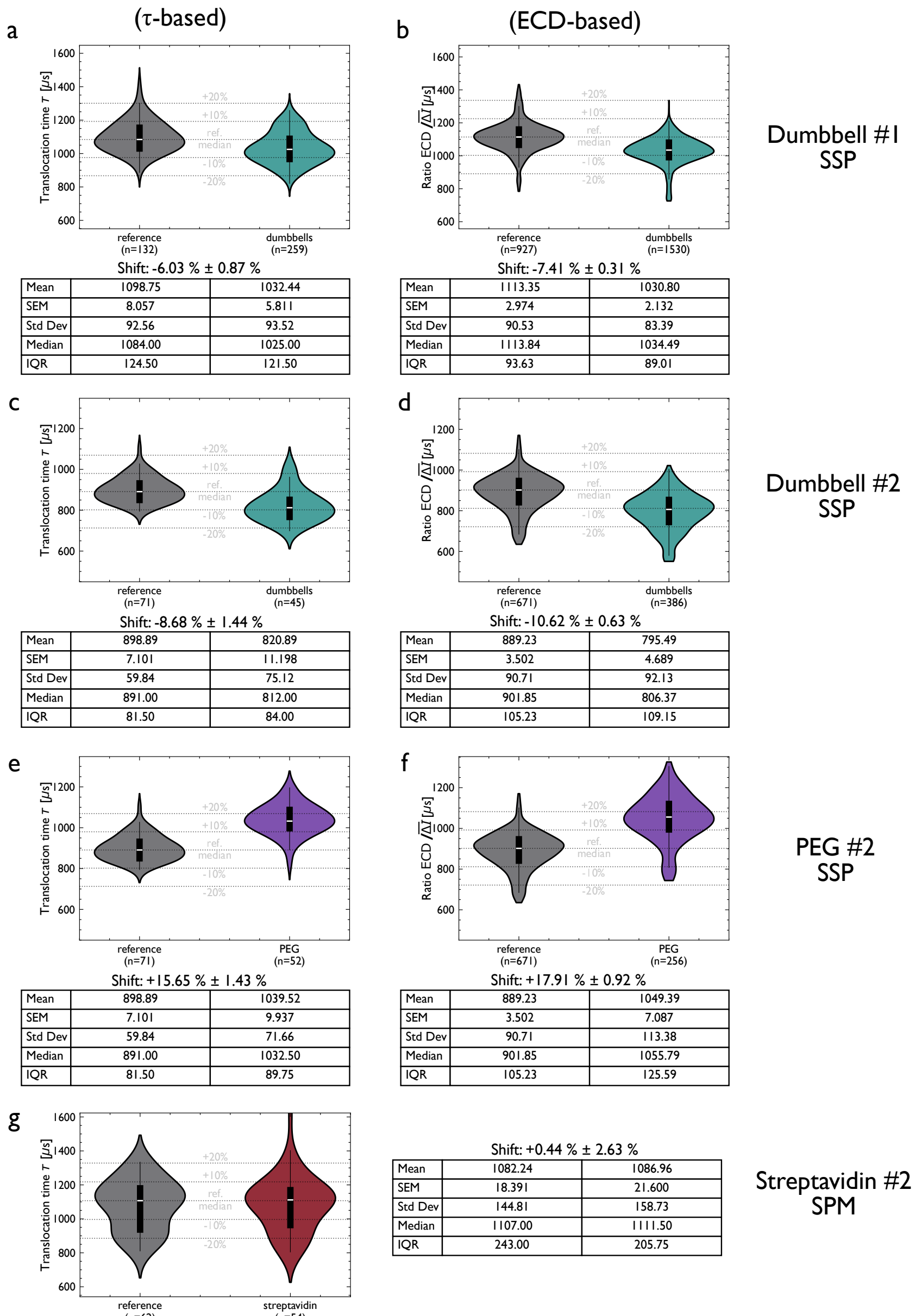

(a) Shift: -6.03 % ± 0.87 %

Mean	1098.75	1032.44
SEM	8.057	5.811
Std Dev	92.56	93.52
Median	1084.00	1025.00
IQR	124.50	121.50

(b) Shift: -7.41 % ± 0.31 %

Mean	1113.35	1030.80
SEM	2.974	2.132
Std Dev	90.53	83.39
Median	1113.84	1034.49
IQR	93.63	89.01

(c) Shift: -8.68 % ± 1.44 %

Mean	898.89	820.89
SEM	7.101	11.198
Std Dev	59.84	75.12
Median	891.00	812.00
IQR	81.50	84.00

(d) Shift: -10.62 % ± 0.63 %

Mean	889.23	795.49
SEM	3.502	4.689
Std Dev	90.71	92.13
Median	901.85	806.37
IQR	105.23	109.15

(e) Shift: +15.65 % ± 1.43 %

Mean	898.89	1039.52
SEM	7.101	9.937
Std Dev	59.84	71.66
Median	891.00	1032.50
IQR	81.50	89.75

(f) Shift: +17.91 % ± 0.92 %

Mean	889.23	1049.39
SEM	3.502	7.087
Std Dev	90.71	113.38
Median	901.85	1055.79
IQR	105.23	125.59

(g) Shift: +0.44 % ± 2.63 %

Mean	1082.24	1086.96
SEM	18.391	21.600
Std Dev	144.81	158.73
Median	1107.00	1111.50
IQR	243.00	205.75

Figure S5: Absolute data control measurements, τ-based analysis on the left and ECD-based on the right (not for streptaividin as not possible for SPM). (a+b) Dumbbell experiment #1 used for Figure 2. (a) τ-based analysis, shows mean decrease of 6%. (b) ECD-based shows mean decreases of 7.4%. (c+d) Dumbbell experiment #2. (c) τ-based analysis. (d) ECD-based analysis. (e+f) PEG experiment #2. (e) τ-based analysis. (f) ECD-based analysis. (g) Streptavidin experiment #2. τ-based analysis. Comparing the mean values between a+b, c+d, e+f, respectively, we observe that the shifts observed with the ECD-based analysis method are slightly higher than that of the $\tau-$based analysis, but the differences are all $\lesssim 2\%$. Comparing the results of a with c, and g with SI-4(c), we observe changes of up to 3 percent between two measurements of same type and analysis method. (a) identical to SI-4(b), (c) identical to SI-8(b).

SI-6 Control 2: Comparison single-pore multiplexing vs. sequential single-pore measurement

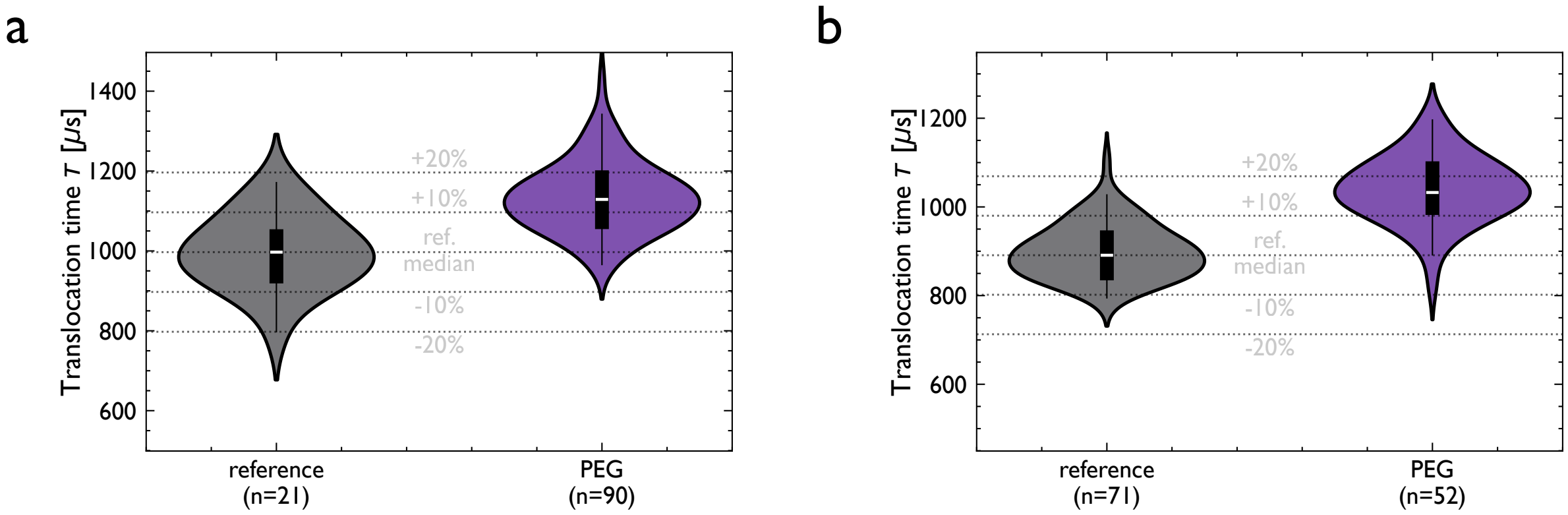

Shift: 13.56 % ± 2.38 %

Mean	997.95	1133.32
SEM	19.385	8.992
Std Dev	88.83	85.30
Median	997.00	1129.00
IQR	101.00	112.50

Shift: 15.65 % ± 1.43 %

Mean	898.89	1039.52
SEM	7.101	9.937
Std Dev	59.84	71.66
Median	891.00	1032.50
IQR	81.50	89.75

Figure S6: (a) Single-pore-multiplexing (SPM, same pore, same time) of PEG, showing a slowdown of $\sim 13\%$ comparing both means and medians. (b) Sequential single-pore measurements (SSP) of the same sample show a similar slow down ($\sim 14\%$) when comparing means and medians. This indicates that our efforts to mitigate evaporation and baseline drift had the desired effect.

SI-7 Control 3: Voltage dependency

(all dumbbells and τ-based)

a

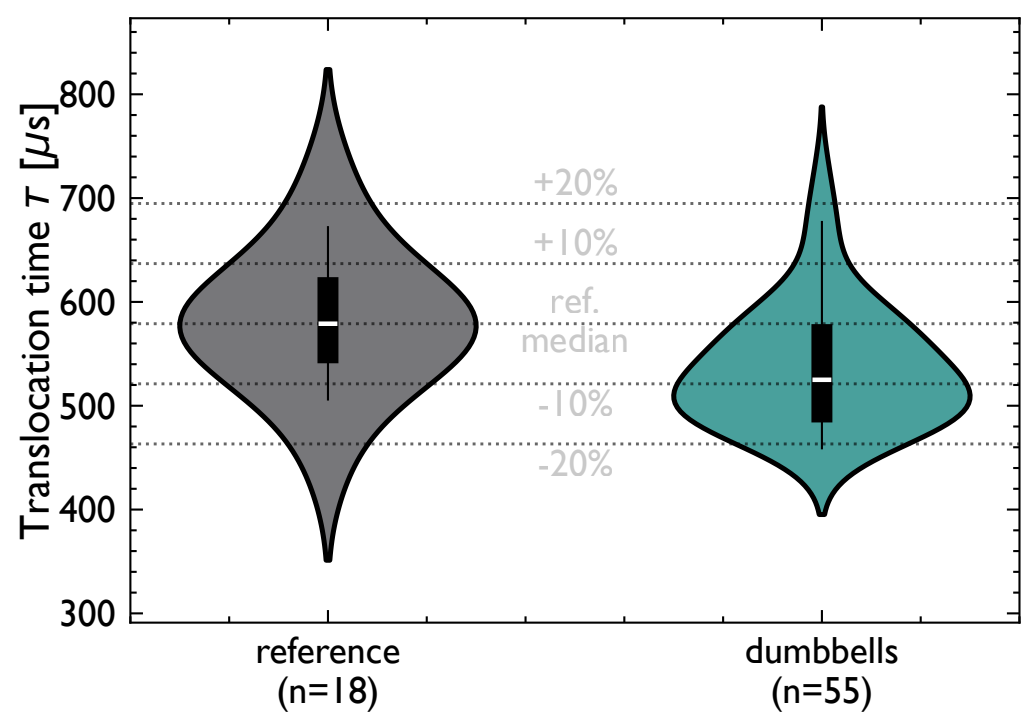

Shift: -9.33%±2.71%

Mean	582.17	536.93
SEM	15.596	7.666
Std Dev	66.17	56.85
Median	579.00	525.00
IQR	62.75	74.50

900 mV

b

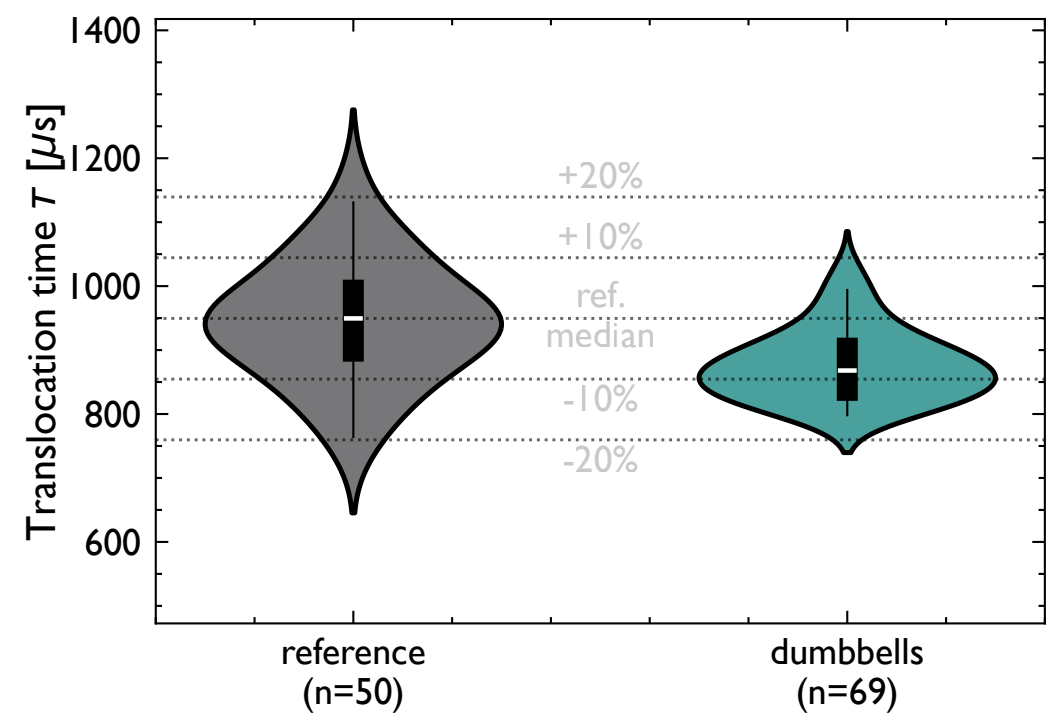

Shift: -8.58%±1.41%

Mean	945.14	875.22
SEM	12.936	6.548
Std Dev	91.47	54.39
Median	949.50	868.00
IQR	95.50	66.00

600 mV

c

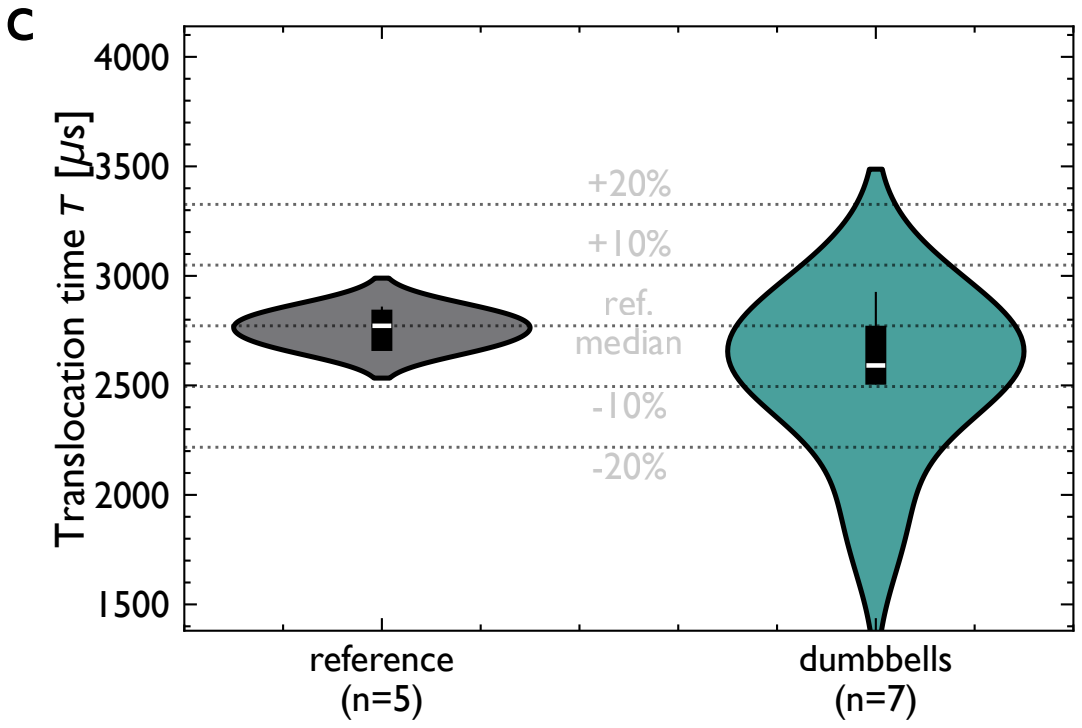

Shift: -6.53%±4.33%

Mean	2759.60	2564.29
SEM	33.242	126.095
Std Dev	74.33	333.62
Median	2772.00	2591.00
IQR	93.00	173.50

300 mV

Figure S7: Sequential single-pore measurements (SSP) for three different voltages: (a) 900 mV, (b) 600 mV, (c) 300 mV. The absolute translocation time increases when lowering the voltage, in line with previous results (Brauburger and Keyser, *Physics of Life Reviews* 2026, 57, 85–131). However, the relative shift in between dumbbell and reference carrier appears to be somewhat preserved. At 300 mV, very few translocations were recorded due to the low electrophoretic force.

SI-8 Control 4: Bigger pore diameters

(all dumbbells and τ-based)

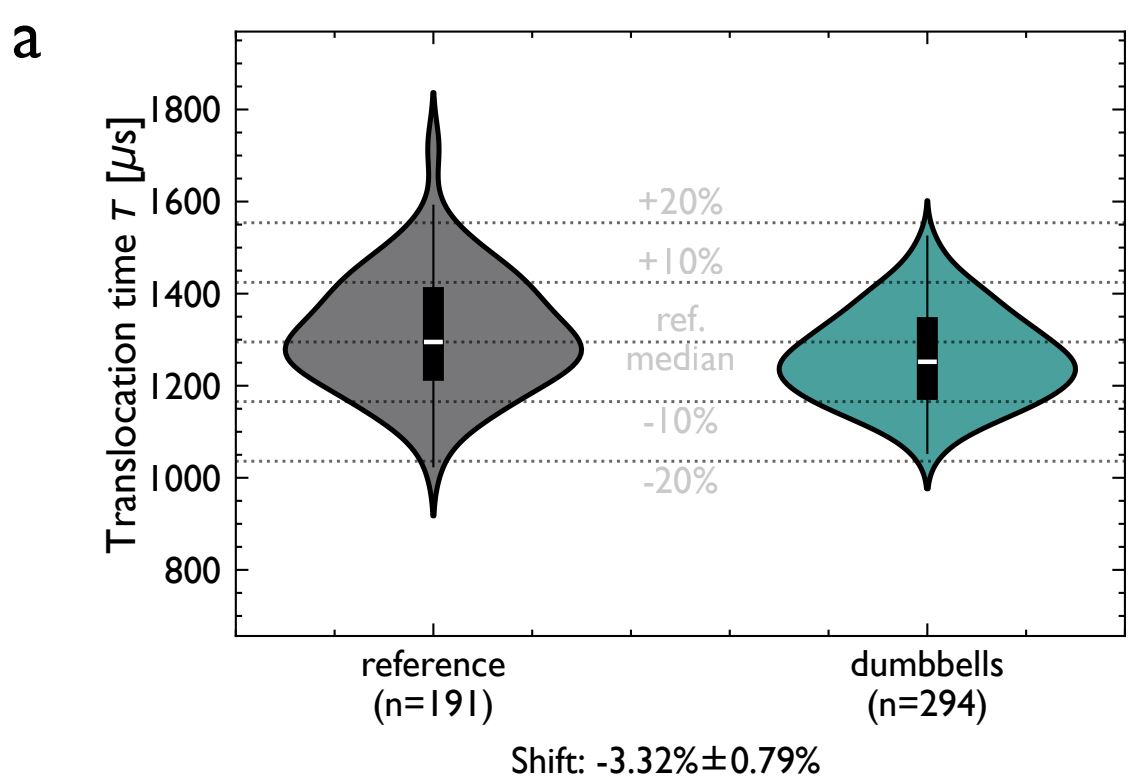

Mean	1312.81	1261.01
SEM	8.908	5.658
Std Dev	123.11	97.02
Median	1295.00	1252.00
IQR	158.00	134.25

big pore 1 (*d* ~ 17.3 nm)

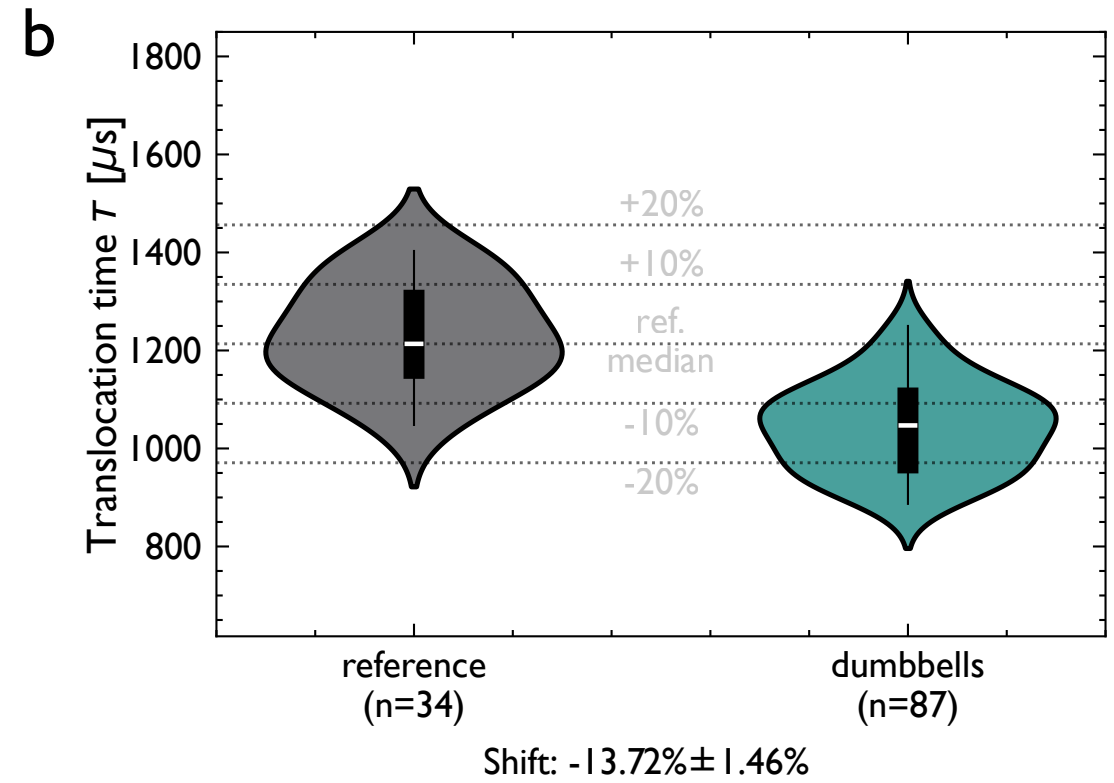

Mean	1233.35	1043.06
SEM	17.553	9.547
Std Dev	102.35	89.05
Median	1213.50	1047.00
IQR	139.00	132.50

big pore 2 (*d* ~ 17.2 nm)

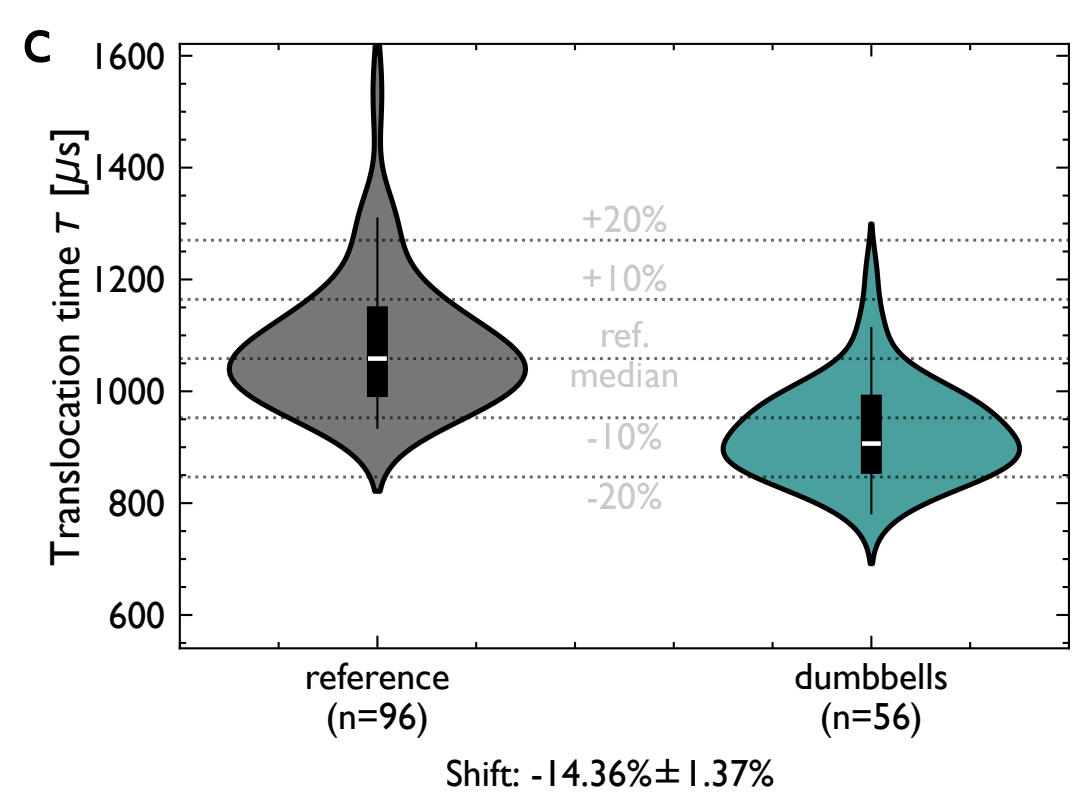

Mean	1080.83	926.12
SEM	11.579	10.795
Std Dev	113.45	80.78
Median	1058.50	906.50
IQR	124.75	104.25

big pore 3 (*d* ~ 14.1 nm)

Figure S8: Sequential single-pore measurements (SSP) for three pores of diameter 14-17%. The maximum observed shifts (-14%) significantly exceed those reported for smaller pores. However, pore 1 also shows a shift of -3%, indicating significant pore-to-pore variations. The larger pores used here also had larger current variations (see Table S1) throughout the measurements, which could correlate with the larger variations shifts. In any case, the overall trend of dumbbell labels speeding up the carrier is also observed here.

SI-9 FWHM definition

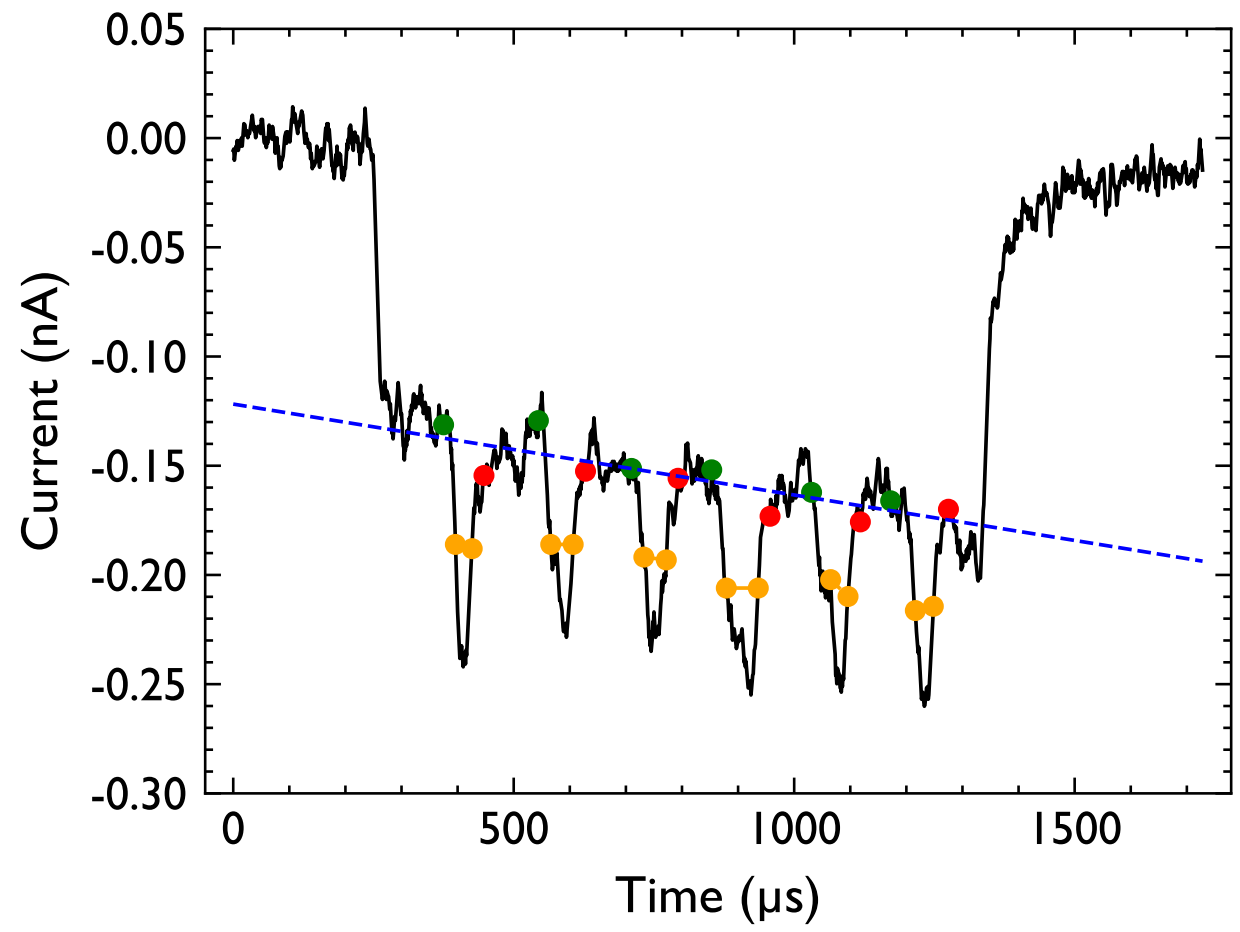

Figure S9: Exemplary trace with fitted baseline for FWHM calculation. The peak values are determined via the scipy.find_peaks python function, the guess of the initial width is then refined by moving outward from the spike minimum in both directions on a smoothed trace and detecting sustained gradient sign changes, obtaining the left/start (green) and right/stop (red) indices for each of the 6 spikes. Then a linear curve is fit through these 12 points (blue dashed). The "half maximum" (in this case rather half mininimum) value for each spike is then in between the minimum y-value of that spike and the y-value of the linear curve at the corresponding x-value. Again moving outward from the peak minimum, the last indices on the right and left side below this value on the side of the minimum are selected. The difference between the right and left index corresponds to the full width at half maximum.

SI-10 Current spike properties

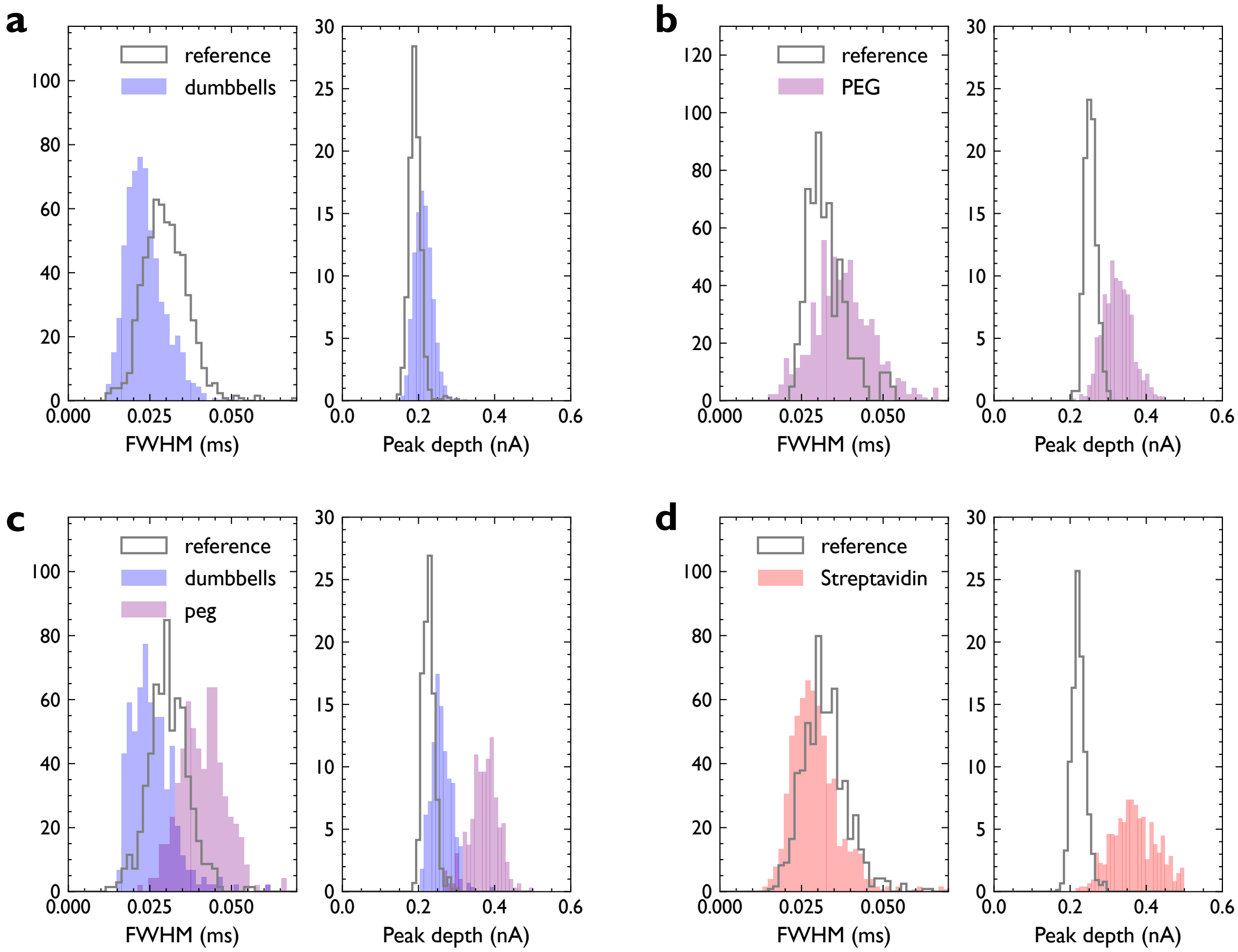

Figure S10: FWHM and spike/peak depth histograms. (a) Dumbbell experiment 1, SSP. (b) PEG experiment 1, SPM. (c) Dumbbell experiment 2, PEG experiment 2, SSP (same pore/reference). (d) Streptavidin experiment 1.

SI-11 Selection examples

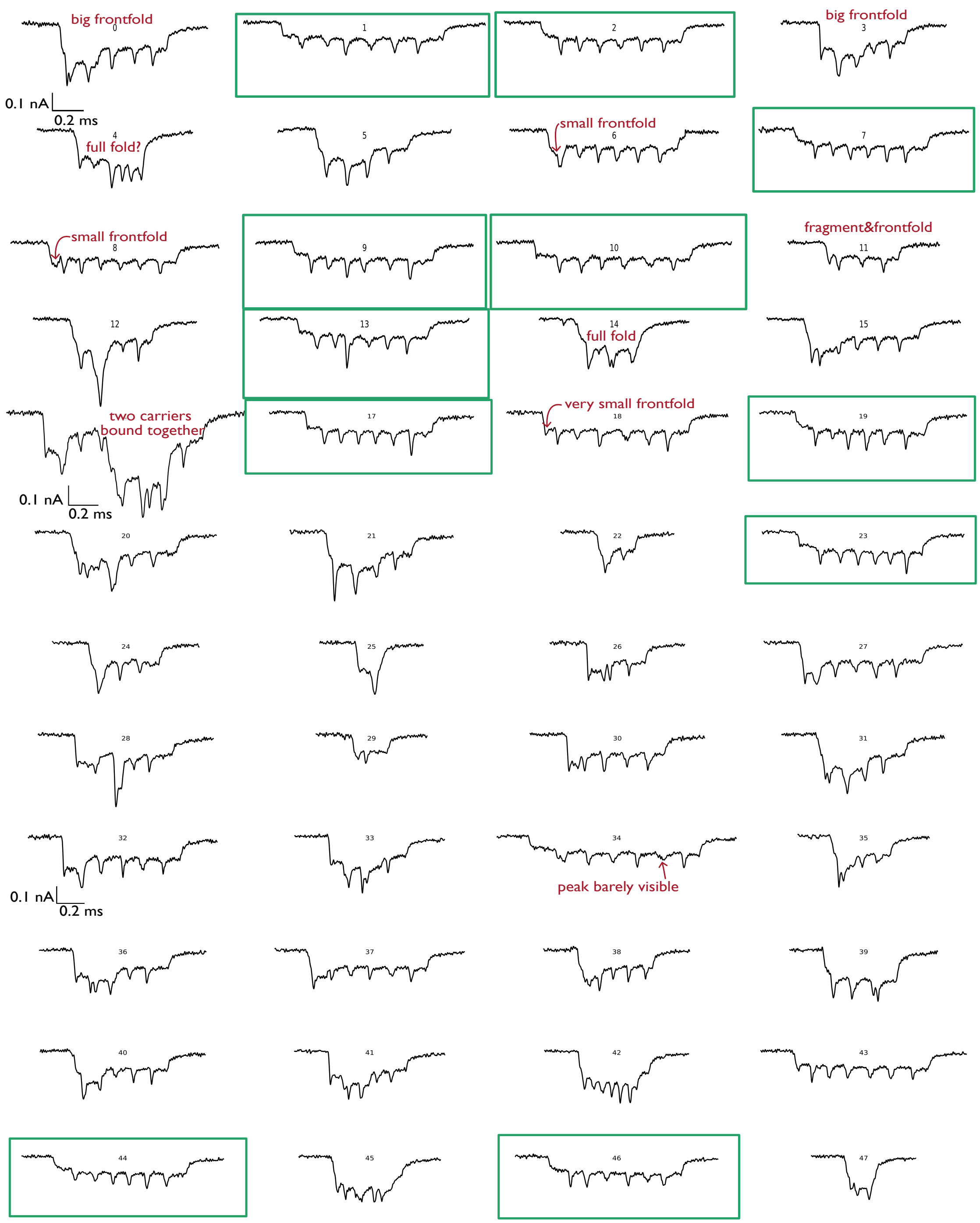

Figure S11: The first 48 traces of the dumbbell measurement in Figure 2. The traces that were considered as fully unfolded are highlighted with a green rectangle. We were very strict on the exclusion, as can be seen for e.g. trace 6 and 18, where the fold at the front are very small. By excluding even small frontfolds, we aim to get a more accurate measure of the translocation velocity for fully unfolded events.

SI-12 Nanopore diameters (Table S1)

Table S1: Summary of pore measurements including resistances, estimated diameters and open current levels at the start and stop of the measurement. The resistances, where possible, were inferred by I-V curves. In some cases (highlighted grey) this data was not recorded and they were obtained via the open-pore current level at the start of the measurements I_{start}. The diameters were inferred using the resistance formula with previously determined geometric parameters from the SI of a previous publication (Bell et al., J. Am. Chem. Soc. 2015, 137, 5, 2035-2041) :

$$R_{\text{pore}} = \underbrace{\frac{4L_1}{\kappa\pi d\,(d + 2L_1\tan\theta_1)}}_{R_{\text{channel}}} + \underbrace{\frac{2}{\kappa\pi\,(d + 2L_1\tan\theta_1)\tan\theta_2}}_{R^{\text{cis}}_{\text{access}}} + \underbrace{\frac{1}{2\kappa d}}_{R^{\text{trans}}_{\text{access}}}\ . \tag{4}$$

The geometric and electrolyte parameters used, as previously, were $L = 100\,\text{nm}$, $\theta_i' = 0.092$, $\theta_i'' = 0.046$, and $\kappa = 16.5\,\text{S}\,\text{m}^{-1}$. In some experiments, an increase of the current baseline I over the course of the measurement is present, which often happens in nanopore experiments. However, at least for the smaller pore measurements, the relative changes are relatively small and reset after exchanging the buffer/sample. For the bigger pore measurements, the differences in current level (while the nominally applied voltage was kept constant) might explain the bigger variations in τ-shifts observed in Figure S9. Single-pore multiplexing (SPM) measurements are denoted as such, the other ones are all sequential single-pore (SSP).

Experiment	Sample loaded	R (MΩ)	d (nm)	I_{start} (nA)	I_{stop} (nA)
Dumbbells Exp.1 (reference)	reference	65.2	8.99	9.2	10.2
Dumbbells Exp.1	dumbbells	65.2	8.99	9.2	9.6
PEG Exp.1 (SPM)	reference&PEG	64.4	9.12	9.8	9.95
Streptavidin Exp.1 (SPM)	reference&Strept.	56.3	10.70	12.4	13.2
Streptavidin Exp.2 (SPM)	reference&Strept.	49.6	12.46	12.1	12.4
Dumbbells & PEG Exp.2 (ref)	reference	69.89	8.28	7.65	7.65
Dumbbells Exp.2	dumbbells	69.89	8.28	7.85	7.95
PEG Exp.2	PEG	69.89	8.28	8.05	8.08
big pore 1	reference	37.7	17.3	13.6	14.6
big pore 1	dumbbells	37.7	17.3	14.9	14.5
big pore 2	reference	37.9	17.2	15.09	15.5
big pore 2	dumbbells	37.9	17.2	14	14.4
big pore 3	reference	44.77	14.1	12.7	13
big pore 3	dumbbells	44.77	14.1	10.9	10.7
voltage control	reference	69.90	8.28	depends on ΔV	
(multiple measurements each)	dumbbells	69.90	8.28	depends on ΔV	

SI-13 Oligonucleotides used for hybridization (Tables S2 to S5)

Table S2: Oligo pool 1: in_between_peaks, corresponding to oligos hybridizing the "complementary Oligonucleotides" in Figure 1(b). The shorter oligos every 20-30 oligos correspond to the last one before the label region

ID	Sequence
in_between_peaks_1	CGTAATCATGGTCATAGCTGTTTCCTGTGTGAAATTGTTA
in_between_peaks_2	TCCGCTCACAATTCCACACAACATACGAGCCGGAAGCATA
in_between_peaks_3	AAGTGTAAAGCCTGGGGTGCCTAATGAGTGAGCTAACTCA
in_between_peaks_4	CATTAATTGCGTTGCGCTCACTGCCCGCTTTCCAGTCGGG
in_between_peaks_5	AAACCTGTCGTGCCAGCTGCATTAATGAATCGGCCAACGC
in_between_peaks_6	GCGGGGAGAGGCGGTTTGCGTATTGGGCGCCAGGGTGGTT
in_between_peaks_7	TTTCTTTTCACCAGTGAGACGGGCAACAGCTGATTGCCCT
in_between_peaks_8	TCACCGCCTGGCCCTGAGAGAGTTGCAGCAAGCGGTCCAC
in_between_peaks_9	GCTGGTTTGCCCCAGCAGGCGAAAATCCTGTTTGATGGTG
in_between_peaks_10	GTTCCGAAATCGGCAAAATCCCTTATAAATCAAAAGAATA
in_between_peaks_11	GCCCGAGATAGGGTTGAGTGTTGTTCCAGTTTGGAACAAG
in_between_peaks_12	AGTCCACTATTAAAGAACGTGGACTCCAACGTCAAAGGGC
in_between_peaks_13	GAAAAACCGTCTATCAGGGCGATGGCCCACTACGTGAACC
in_between_peaks_14	ATCACCCAAATCAAGTTTTTTGGGGTCGAGGTGCCGTAAA
in_between_peaks_15	GCACTAAATCGGAACCCTAAAGGGAGCCCCCGATTTAGAG
in_between_peaks_16	CTTGACGGGGAAAGCCGGCGAACGTGGCGAGAAAGGAAGG
in_between_peaks_17	GAAGAAAGCGAAAGGAGCGGGCGCTAGGGCGCTGGCAAGT
in_between_peaks_18	GTAGCGGTCACGCTGCGCGTAACCACCACACCCGCCGCGC
in_between_peaks_19	TTAATGCGCCGCTACAGGGCGCGTACTATGGTTGCTTTGA
in_between_peaks_20	CGAGCACGTATAACGTGCTTTCCTCGTTAGAATCAGAGCG
in_between_peaks_21	GGAGCTAAACAGGAGGCCGATTAAAGGGATTTTAGACAGG
in_between_peaks_22	AACGGTACGCCAGAATCCTGAGAAGTGTTTTTATAATCAG
in_between_peaks_23	TGAGGCCACCGAGTAAAAGAGTCTGTCCATCACGCAAATT
in_between_peaks_24	AACCGTTGTAGCAATACTTCTTTGATTAGTAATAACATCA
in_between_peaks_25	CTTGCCTGAGTAGAAGAACTCAAACTATCGGCCTTGCTGG
in_between_peaks_26	TAATATCCAGAACAATATTACCGCCAGCCATTGCAACAGG
in_between_peaks_27	AAAAACGCTCATGGAAATACCTACATTTTGACGCTCAATC
in_between_peaks_28	GTCTGAAATGGATTATTTACATT
in_between_peaks_29	CCAGCAGCAAATGAAAAATCTAAAGCATCACCTTGCTGAA
in_between_peaks_30	CCTCAAATATCAAACCCTCAATCAATATCTGGTCAGTTGG
in_between_peaks_31	CAAATCAACAGTTGAAAGGAATTGAGGAAGGTTATCTAAA
in_between_peaks_32	ATATCTTTAGGAGCACTAACAACTAATAGATTAGAGCCGT
in_between_peaks_33	CAATAGATAATACATTTGAGGATTTAGAAGTATTAGACTT
in_between_peaks_34	TACAAACAATTCGACAACTCGTATTAAATCCTTTGCCCGA
in_between_peaks_35	ACGTTATTAATTTTAAAAGTTTGAGTAACATTATCATTTT
in_between_peaks_36	GCGGAACAAAGAAACCACCAGAAGGAGCGGAATTATCATC
in_between_peaks_37	ATATTCCTGATTATCAGATGATGGCAATTCATCAATATAA
in_between_peaks_38	TCCTGATTGTTTGGATTATACTTCTGAATAATGGAAGGGT
in_between_peaks_39	TAGAACCTACCATATCAAAATTATTTGCACGTAAAACAGA
in_between_peaks_40	AATAAAGAAATTGCGTAGATTTTCAGGTTTAACGTCAGAT
in_between_peaks_41	GAATATACAGTAACAGTACCTTTTACATCGGGAGAAACAA
in_between_peaks_42	TAACGGATTCGCCTGATTGCTTTGAATACCAAGTTACAAA
in_between_peaks_43	ATCGCGCAGAGGCGAATTATTCATTTCAATTACCTGAGCA
in_between_peaks_44	AAAGAAGATGATGAAACAAACATCAAGAAAACAAAATTAA
in_between_peaks_45	TTACATTTAACAATTTCATTTGAATTACCTTTTTTAATGG
in_between_peaks_46	AAACAGTACATAAATCAATATATGTGAGTGAATAACCTTG
in_between_peaks_47	CTTCTGTAAATCGTCGCTATTAATTAATTT
in_between_peaks_48	TGTGATAAATAAGGCGTTAAATAAGAATAAACACCGGAAT

Continued on next page...

Table S2: (continued)

ID	Sequence
in_between_peaks_49	CATAATTACTAGAAAAAGCCTGTTTAGTATCATATGCGTT
in_between_peaks_50	ATACAAATTCTTACCAGTATAAAGCCAACGCTCAACAGTA
in_between_peaks_51	GGGCTTAATTGAGAATCGCCATATTTAACAACGCCAACAT
in_between_peaks_52	GTAATTTAGGCAGAGGCATTTTCGAGCCAGTAATAAGAGA
in_between_peaks_53	ATATAAAGTACCGACAAAAGGTAAAGTAATTCTGTCCAGA
in_between_peaks_54	CGACGACAATAAACAACATGTTCAGCTAATGCAGAACGCG
in_between_peaks_55	CCTGTTTATCAACAATAGATAAGTCCTGAACAAGAAAAAT
in_between_peaks_56	AATATCCCATCCTAATTTACGAGCATGTAGAAACCAATCA
in_between_peaks_57	ATAATCGGCTGTCTTTCCTTATCATTCCAAGAACGGGTAT
in_between_peaks_58	TAAACCAAGTACCGCACTCATCGAGAACAAGCAAGCCGTT
in_between_peaks_59	TTTATTTTCATCGTAGGAATCATTACCGCGCCCAATAGCA
in_between_peaks_60	AGCAAATCAGATATAGAAGGCTTATCCGGTATTCTAAGAA
in_between_peaks_61	CGCGAGGCGTTTTAGCGAACCTCCCGACTTGCGGGAGGTT
in_between_peaks_62	TTGAAGCCTTAAATCAAGATTAGTTGCTATTTTGCACCCA
in_between_peaks_63	GCTACAATTTTATCCTGAATCTTACCAACGCTAACGAGCG
in_between_peaks_64	TCTTTCCAGAGCCTAATTTGCCAGTTACAAAATAAACAGC
in_between_peaks_65	CATATTATTTATCCCAATCCAAATAAGAAACGATTTTTTG
in_between_peaks_66	TTTAACGTCAAAAATGAAAATAGCAGCCTT
in_between_peaks_67	GGAATACCCAAAAGAACTGGCATGATTAAGACTCCTTATT
in_between_peaks_68	ACGCAGTATGTTAGCAAACGTAGAAAATACATACATAAAG
in_between_peaks_69	GTGGCAACATATAAAAGAAACGCAAAGACACCACGGAATA
in_between_peaks_70	AGTTTATTTTGTCACAATCAATAGAAAATTCATATGGTTT
in_between_peaks_71	ACCAGCGCCAAAGACAAAAGGGCGACATTCAACCGATTGA
in_between_peaks_72	GGGAGGGAAGGTAAATATTGACGGAAATTATTCATTAAAG
in_between_peaks_73	GTGAATTATCACCGTCACCGACTTGAGCCATTTGGGAATT
in_between_peaks_74	AGAGCCAGCAAAATCACCAGTAGCACCATTACCATTAGCA
in_between_peaks_75	AGGCCGGAAACGTCACCAATGAAACCATCGATAGCAGCAC
in_between_peaks_76	CGTAATCAGTAGCGACAGAATCAAGTTTGCCTTTAGCGTC
in_between_peaks_77	AGACTGTAGCGCGTTTTCATCGGCATTTTCGGTCATAGCC
in_between_peaks_78	CCCTTATTAGCGTTTGCCATCTTTTCATAATCAAAATCAC
in_between_peaks_79	CGGAACCAGAGCCACCACCGGAACCGCCTCCCTCAGAGCC
in_between_peaks_80	GCCACCCTCAGAACCGCCACCCTCAGAGCCACCACCCTCA
in_between_peaks_81	GAGCCGCCACCAGAACCACCACCAGAGCCGCCGCCAGCAT
in_between_peaks_82	TGACAGGAGGTTGAGGCAGGTCAGACGATTGGCCTTGATA
in_between_peaks_83	TTCACAAACAAATAAATCCTCATTAAAGCCAGAATGGAAA
in_between_peaks_84	GCGCAGTCTCTGAATTTACCGTTCCAGTAAGCGTCATACA
in_between_peaks_85	TGGCTTTTGATGATACAGGAGTGTACTGGT
in_between_peaks_86	AGAACCGCCACCCTCAGAACCGCCACCCTCAGAGCCACCA
in_between_peaks_87	CCCTCATTTTCAGGGATAGCAAGCCCAATAGGAACCCATG
in_between_peaks_88	TACCGTAACACTGAGTTTCGTCACCAGTACAAACTACAAC
in_between_peaks_89	GCCTGTAGCATTCCACAGACAGCCCTCATAGTTAGCGTAA
in_between_peaks_90	CGATCTAAAGTTTTGTCGTCTTTCCAGACGTTAGTAAATG
in_between_peaks_91	AATTTTCTGTATGGGATTTTGCTAAACAACTTTCAACAGT
in_between_peaks_92	TTCAGCGGAGTGAGAATAGAAAGGAACAACTAAAGGAATT
in_between_peaks_93	GCGAATAATAATTTTTTCACGTTGAAAATCTCCAAAAAAA
in_between_peaks_94	AGGCTCCAAAAGGAGCCTTTAATTGTATCGGTTTATCAGC
in_between_peaks_95	TTGCTTTCGAGGTGAATTTCTTAAACAGCTTGATACCGAT
in_between_peaks_96	AGTTGCGCCGACAATGACAACAACCATCGCCCACGCATAA
in_between_peaks_97	CCGATATATTCGGTCGCTGAGGCTTGCAGGGAGTTAAAGG
in_between_peaks_98	CCGCTTTTGCGGGATCGTCACCCTCAGCAGCGAAAGACAG
in_between_peaks_99	CATCGGAACGAGGGTAGCAACGGCTACAGAGGCTTTGAGG
in_between_peaks_100	ACTAAAGACTTTTTCATGAGGAAGTTTCCATTAAACGGGT
in_between_peaks_101	AAAATACGTAATGCCACTACGAAGGCACCAACCTAAAACG

Continued on next page...

Table S2: (continued)

ID	Sequence
in_between_peaks_102	AAAGAGGCAAAAGAATACACTAAAACACTCATCTTTGACC
in_between_peaks_103	CCCAGCGATTATACCAAGCGCGAAACAAAGTACAACGGAG
in_between_peaks_104	ATTTGTATCATCGCCTGATAAATTGTGTCG
in_between_peaks_105	TGGGCTTGAGATGGTTTAATTTCAACTTTAATCATTGTGA
in_between_peaks_106	ATTACCTTATGCGATTTTAAGAACTGGCTCATTATACCAG
in_between_peaks_107	TCAGGACGTTGGGAAGAAAAATCTACGTTAATAAAACGAA
in_between_peaks_108	CTAACGGAACAACATTATTACAGGTAGAAAGATTCATCAG
in_between_peaks_109	TTGAGATTTAGGAATACCACATTCAACTAATGCAGATACA
in_between_peaks_110	TAACGCCAAAAGGAATTACGAGGCATAGTAAGAGCAACAC
in_between_peaks_111	TATCATAACCCTCGTTTACCAGACGACGATAAAAACCAAA
in_between_peaks_112	ATAGCGAGAGGCTTTTGCAAAAGAAGTTTTGCCAGAGGGG
in_between_peaks_113	GTAATAGTAAAATGTTTAGACTGGATAGCGTCCAATACTG
in_between_peaks_114	CGGAATCGTCATAAATATTCATTGAATCCCCCTCAAATGC
in_between_peaks_115	TTTAAACAGTTCAGAAAACGAGAATGACCATAAATCAAAA
in_between_peaks_116	ATCAGGTCTTTACCCTGACTATTATAGTCAGAAGCAAAGC
in_between_peaks_117	GGATTGCATCAAAAAGATTAAGAGGAAGCCCGAAAGACTT
in_between_peaks_118	CAAATATCGCGTTTTAATTCGAGCTTCAAAGCGAACCAGA
in_between_peaks_119	CCGGAAGCAAACTCCAACAGGTCAGGATTAGAGAGTACCT
in_between_peaks_120	TTAATTGCTCCTTTTGATAAGAGGTCATTTTTGCGGATGG
in_between_peaks_121	CTTAGAGCTTAATTGCTGAATATAATGCTGTAGCTCAACA
in_between_peaks_122	TGTTTTAAATATGCAACTAAAGTACGGTGTCTGGAAGTTT
in_between_peaks_123	CATTCCATATAACAGTTGATTCCCAATTCT
in_between_peaks_124	AGCCTTTATTTCAACGCAAGGATAAAAATTTTTAGAACCC
in_between_peaks_125	TCATATATTTTAAATGCAATGCCTGAGTAATGTGTAGGTA
in_between_peaks_126	AAGATTCAAAAGGGTGAGAAAGGCCGGAGACAGTCAAATC
in_between_peaks_127	ACCATCAATATGATATTCAACCGTTCTAGCTGATAAATTA
in_between_peaks_128	ATGCCGGAGAGGGTAGCTATTTTTGAGAGATCTACAAAGG
in_between_peaks_129	CTATCAGGTCATTGCCTGAGAGTCTGGAGCAAACAAGAGA
in_between_peaks_130	ATCGATGAACGGTAATCGTAAAACTAGCATGTCAATCATA
in_between_peaks_131	TGTACCCCGGTTGATAATCAGAAAAGCCCCAAAAACAGGA
in_between_peaks_132	AGATTGTATAAGCAAATATTTAAATTGTAAACGTTAATAT
in_between_peaks_133	TTTGTTAAAATTCGCATTAAATTTTTGTTAAATCAGCTCA
in_between_peaks_134	TTTTTTAACCAATAGGAACGCCATCAAAAATAATTCGCGT
in_between_peaks_135	CTGGCCTTCCTGTAGCCAGCTTTCATCAACATTAAATGTG
in_between_peaks_136	AGCGAGTAACAACCCGTCGGATTCTCCGTGGGAACAAACG
in_between_peaks_137	GCGGATTGACCGTAATGGGATAGGTCACGTTGGTGTAGAT
in_between_peaks_138	GGGCGCATCGTAACCGTGCATCTGCCAGTTTGAGGGGACG
in_between_peaks_139	ACGACAGTATCGGCCTCAGGAAGATCGCACTCCAGCCAGC
in_between_peaks_140	TTTCCGGCACCGCTTCTGGTGCCGGAAACCAGGCAAAGCG
in_between_peaks_141	CCATTCGCCATTCAGGCTGCGCAACTGTTGGGAAGGGCGA
in_between_peaks_142	TCGGTGCGGGCCTCTTCGCTATTACGCCAGCTGGCGAAAG
in_between_peaks_143	GGGGATGTGCTGCAAGGCGATTAAGTTGGGTAACGCCAGG
in_between_peaks_144	GTTTTCCCAGTCACGACGTTGTAAAACGACGGCCAGTGCC
in_between_peaks_145	AAGCTTGCATGCCTGCAGGTCGACTCTAGAGGATC

Table S3: Oligo pool 2: dsDNA_nopeaks, corresponding to oligos hybridizing the labelled regions for dsDNA carriers (10 oligos per label region).

ID	Sequence
dsDNA_nopeaks_1	GGCAGATTCACCAGTCACACGACCA
dsDNA_nopeaks_2	GTAATAAAAGGGACATTCTGGCCAA
dsDNA_nopeaks_3	CAGAGATAGAACCCTTCTGACCTGA

Continued on next page...

Table S3: (continued)

ID	Sequence
dsDNA__nopeaks__4	AAGCGTAAGAATACGTGGCACAGAC
dsDNA__nopeaks__5	AATATTTTTGAATGGCTATTAGTCT
dsDNA__nopeaks__6	TTAATGCGCGAACTGATAGCCCTAA
dsDNA__nopeaks__7	AACATCGCCATTAAAAATACCGAAC
dsDNA__nopeaks__8	GAACCACCAGCAGAAGATAAAACAG
dsDNA__nopeaks__9	AGGTGAGGCGGTCAGTATTAACACC
dsDNA__nopeaks__10	GCCTGCAACAGTGCCACGCTGAGAG
dsDNA__nopeaks__11	TCCCTTAGAATCCTTGAAAACATAG
dsDNA__nopeaks__12	CGATAGCTTAGATTAAGACGCTGAG
dsDNA__nopeaks__13	AAGAGTCAATAGTGAATTTATCAAA
dsDNA__nopeaks__14	ATCATAGGTCTGAGAGACTACCTTT
dsDNA__nopeaks__15	TTAACCTCCGGCTTAGGTTGGGTTA
dsDNA__nopeaks__16	TATAACTATATGTAAATGCTGATGC
dsDNA__nopeaks__17	AAATCCAATCGCAAGACAAAGAACG
dsDNA__nopeaks__18	CGAGAAAACTTTTTCAAATATATTT
dsDNA__nopeaks__19	TAGTTAATTTCATCTTCTGACCTAA
dsDNA__nopeaks__20	ATTTAATGGTTTGAAATACCGACCG
dsDNA__nopeaks__21	TACAGAGAGAATAACATAAAAACAG
dsDNA__nopeaks__22	GGAAGCGCATTAGACGGGAGAATTA
dsDNA__nopeaks__23	ACTGAACACCCTGAACAAAGTCAGA
dsDNA__nopeaks__24	GGGTAATTGAGCGCTAATATCAGAG
dsDNA__nopeaks__25	AGATAACCCACAAGAATTGAGTTAA
dsDNA__nopeaks__26	GCCCAATAATAAGAGCAAGAAACAA
dsDNA__nopeaks__27	TGAAATAGCAATAGCTATCTTACCG
dsDNA__nopeaks__28	AAGCCCTTTTTAAGAAAAGTAAGCA
dsDNA__nopeaks__29	GATAGCCGAACAAAGTTACCAGAAG
dsDNA__nopeaks__30	GAAACCGAGGAAACGCAATAATAAC
dsDNA__nopeaks__31	AATAAGTTTTAACGGGGTCAGTGCC
dsDNA__nopeaks__32	TTGAGTAACAGTGCCCGTATAAACA
dsDNA__nopeaks__33	GTTAATGCCCCCTGCCTATTTCGGA
dsDNA__nopeaks__34	ACCTATTATTCTGAAACATGAAAGT
dsDNA__nopeaks__35	ATTAAGAGGCTGAGACTCCTCAAGA
dsDNA__nopeaks__36	GAAGGATTAGGATTAGCGGGGTTTT
dsDNA__nopeaks__37	GCTCAGTACCAGGCGGATAAGTGCC
dsDNA__nopeaks__38	GTCGAGAGGGTTGATATAAGTATAG
dsDNA__nopeaks__39	CCCGGAATAGGTGTATCACCGTACT
dsDNA__nopeaks__40	CAGGAGGTTTAGTACCGCCACCCTC
dsDNA__nopeaks__41	AAATCCGCGACCTGCTCCATGTTAC
dsDNA__nopeaks__42	TTAGCCGGAACGAGGCGCAGACGGT
dsDNA__nopeaks__43	CAATCATAAGGGAACCGAACTGACC
dsDNA__nopeaks__44	AACTTTGAAAGAGGACAGATGAACG
dsDNA__nopeaks__45	GTGTACAGACCAGGCGCATAGGCTG
dsDNA__nopeaks__46	GCTGACCTTCATCAAGAGTAATCTT
dsDNA__nopeaks__47	GACAAGAACCGGATATTCATTACCC
dsDNA__nopeaks__48	AAATCAACGTAACAAAGCTGCTCAT
dsDNA__nopeaks__49	TCAGTGAATAAGGCTTGCCCTGACG
dsDNA__nopeaks__50	AGAAACACCAGAACGAGTAGTAAAT
dsDNA__nopeaks__51	GCGAACGAGTAGATTTAGTTTGACC
dsDNA__nopeaks__52	ATTAGATACATTTCGCAAATGGTCA
dsDNA__nopeaks__53	ATAACCTGTTTAGCTATATTTTCAT
dsDNA__nopeaks__54	TTGGGGCGCGAGCTGAAAAGGTGGC
dsDNA__nopeaks__55	ATCAATTCTACTAATAGTAGTAGCA
dsDNA__nopeaks__56	TTAACATCCAATAAATCATACAGGC

Continued on next page...

Table S3: (continued)

ID	Sequence
dsDNA_nopeaks_57	AAGGCAAAGAATTAGCAAAATTAAG
dsDNA_nopeaks_58	CAATAAAGCCTCAGAGCATAAAGCT
dsDNA_nopeaks_59	AAATCGGTTGTACCAAAAACATTAT
dsDNA_nopeaks_60	GACCCTGTAATACTTTTGCGGGAGA

Table S4: Oligo pool 3: peaks_dumbbells, corresponding to oligos hybridizing the labelled regions for dumbbell carriers (10 oligos per peak).

ID	Sequence
peaks_dumbbells_1	GGCAGATTCACCtcctcttttgaggaacaagttttcttgtAGTCACACGACCA
peaks_dumbbells_2	GTAATAAAAGGGtcctcttttgaggaacaagttttcttgtACATTCTGGCCAA
peaks_dumbbells_3	CAGAGATAGAACtcctcttttgaggaacaagttttcttgtCCTTCTGACCTGA
peaks_dumbbells_4	AAGCGTAAGAATtcctcttttgaggaacaagttttcttgtACGTGGCACAGAC
peaks_dumbbells_5	AATATTTTTGAAtcctcttttgaggaacaagttttcttgtTGGCTATTAGTCT
peaks_dumbbells_6	TTAATGCGCGAAtcctcttttgaggaacaagttttcttgtCTGATAGCCCTAA
peaks_dumbbells_7	AACATCGCCATTtcctcttttgaggaacaagttttcttgtAAAAATACCGAAC
peaks_dumbbells_8	GAACCACCAGCAtcctcttttgaggaacaagttttcttgtGAAGATAAAACAG
peaks_dumbbells_9	AGGTGAGGCGGTtcctcttttgaggaacaagttttcttgtCAGTATTAACACC
peaks_dumbbells_10	GCCTGCAACAGTtcctcttttgaggaacaagttttcttgtGCCACGCTGAGAG
peaks_dumbbells_11	TCCCTTAGAATCtcctcttttgaggaacaagttttcttgtCTTGAAAACATAG
peaks_dumbbells_12	CGATAGCTTAGAtcctcttttgaggaacaagttttcttgtTTAAGACGCTGAG
peaks_dumbbells_13	AAGAGTCAATAGtcctcttttgaggaacaagttttcttgtTGAATTTATCAAA
peaks_dumbbells_14	ATCATAGGTCTGtcctcttttgaggaacaagttttcttgtAGAGACTACCTTT
peaks_dumbbells_15	TTAACCTCCGGCtcctcttttgaggaacaagttttcttgtTTAGGTTGGGTTA
peaks_dumbbells_16	TATAACTATATGtcctcttttgaggaacaagttttcttgtTAAATGCTGATGC
peaks_dumbbells_17	AAATCCAATCGCtcctcttttgaggaacaagttttcttgtAAGACAAAGAACG
peaks_dumbbells_18	CGAGAAAACTTTtcctcttttgaggaacaagttttcttgtTTCAAATATATTT
peaks_dumbbells_19	TAGTTAATTTCAtcctcttttgaggaacaagttttcttgtTCTTCTGACCTAA
peaks_dumbbells_20	ATTTAATGGTTTtcctcttttgaggaacaagttttcttgtGAAATACCGACCG
peaks_dumbbells_21	TACAGAGAGAATtcctcttttgaggaacaagttttcttgtAACATAAAAACAG
peaks_dumbbells_22	GGAAGCGCATTAtcctcttttgaggaacaagttttcttgtGACGGGAGAATTA
peaks_dumbbells_23	ACTGAACACCCTtcctcttttgaggaacaagttttcttgtGAACAAAGTCAGA
peaks_dumbbells_24	GGGTAATTGAGCtcctcttttgaggaacaagttttcttgtGCTAATATCAGAG
peaks_dumbbells_25	AGATAACCCACAtcctcttttgaggaacaagttttcttgtAGAATTGAGTTAA
peaks_dumbbells_26	GCCCAATAATAAtcctcttttgaggaacaagttttcttgtGAGCAAGAAACAA
peaks_dumbbells_27	TGAAATAGCAATtcctcttttgaggaacaagttttcttgtAGCTATCTTACCG
peaks_dumbbells_28	AAGCCCTTTTTAtcctcttttgaggaacaagttttcttgtAGAAAAGTAAGCA
peaks_dumbbells_29	GATAGCCGAACAtcctcttttgaggaacaagttttcttgtAAGTTACCAGAAG
peaks_dumbbells_30	GAAACCGAGGAAtcctcttttgaggaacaagttttcttgtACGCAATAATAAC
peaks_dumbbells_31	AATAAGTTTTAAtcctcttttgaggaacaagttttcttgtCGGGGTCAGTGCC
peaks_dumbbells_32	TTGAGTAACAGTtcctcttttgaggaacaagttttcttgtGCCCGTATAAACA
peaks_dumbbells_33	GTTAATGCCCCCtcctcttttgaggaacaagttttcttgtTGCCTATTTCGGA
peaks_dumbbells_34	ACCTATTATTCTtcctcttttgaggaacaagttttcttgtGAAACATGAAAGT
peaks_dumbbells_35	ATTAAGAGGCTGtcctcttttgaggaacaagttttcttgtAGACTCCTCAAGA
peaks_dumbbells_36	GAAGGATTAGGAtcctcttttgaggaacaagttttcttgtTTAGCGGGGTTTT
peaks_dumbbells_37	GCTCAGTACCAGtcctcttttgaggaacaagttttcttgtGCGGATAAGTGCC
peaks_dumbbells_38	GTCGAGAGGGTTtcctcttttgaggaacaagttttcttgtGATATAAGTATAG
peaks_dumbbells_39	CCCGGAATAGGTtcctcttttgaggaacaagttttcttgtGTATCACCGTACT
peaks_dumbbells_40	CAGGAGGTTTAGtcctcttttgaggaacaagttttcttgtTACCGCCACCCTC
peaks_dumbbells_41	AAATCCGCGACCtcctcttttgaggaacaagttttcttgtTGCTCCATGTTAC
peaks_dumbbells_42	TTAGCCGGAACGtcctcttttgaggaacaagttttcttgtAGGCGCAGACGGT
peaks_dumbbells_43	CAATCATAAGGGtcctcttttgaggaacaagttttcttgtAACCGAACTGACC

Continued on next page...

Table S4: (continued)

ID	Sequence
peaks_dumbbells_44	AACTTTGAAAGAtcctcttttgaggaacaagttttcttgtGGACAGATGAACG
peaks_dumbbells_45	GTGTACAGACCAtcctcttttgaggaacaagttttcttgtGGCGCATAGGCTG
peaks_dumbbells_46	GCTGACCTTCATtcctcttttgaggaacaagttttcttgtCAAGAGTAATCTT
peaks_dumbbells_47	GACAAGAACCGGtcctcttttgaggaacaagttttcttgtATATTCATTACCC
peaks_dumbbells_48	AAATCAACGTAAtcctcttttgaggaacaagttttcttgtCAAAGCTGCTCAT
peaks_dumbbells_49	TCAGTGAATAAGtcctcttttgaggaacaagttttcttgtGCTTGCCCTGACG
peaks_dumbbells_50	AGAAACACCAGAtcctcttttgaggaacaagttttcttgtACGAGTAGTAAAT
peaks_dumbbells_51	GCGAACGAGTAGtcctcttttgaggaacaagttttcttgtATTTAGTTTGACC
peaks_dumbbells_52	ATTAGATACATTtcctcttttgaggaacaagttttcttgtTCGCAAATGGTCA
peaks_dumbbells_53	ATAACCTGTTTAtcctcttttgaggaacaagttttcttgtGCTATATTTTCAT
peaks_dumbbells_54	TTGGGGCGCGAGtcctcttttgaggaacaagttttcttgtCTGAAAAGGTGGC
peaks_dumbbells_55	ATCAATTCTACTtcctcttttgaggaacaagttttcttgtAATAGTAGTAGCA
peaks_dumbbells_56	TTAACATCCAATtcctcttttgaggaacaagttttcttgtAAATCATACAGGC
peaks_dumbbells_57	AAGGCAAAGAATtcctcttttgaggaacaagttttcttgtTAGCAAAATTAAG
peaks_dumbbells_58	CAATAAAGCCTCtcctcttttgaggaacaagttttcttgtAGAGCATAAAGCT
peaks_dumbbells_59	AAATCGGTTGTAtcctcttttgaggaacaagttttcttgtCCAAAAACATTAT
peaks_dumbbells_60	GACCCTGTAATAtcctcttttgaggaacaagttttcttgtCTTTTGCGGGAGA

Table S5: Oligo pool 4: peaks_adapter, corresponding to oligos hybridizing the labelled regions for reference, PEG and streptavidin carriers (10 oligos per peak).

ID	Sequence
peaks_adapter_1	GGCAGATTCACCAGTCACACGACCAtttggatatcactcattagtggt
peaks_adapter_2	GTAATAAAAGGGACATTCTGGCCAAtttggatatcactcattagtggt
peaks_adapter_3	CAGAGATAGAACCCTTCTGACCTGAtttggatatcactcattagtggt
peaks_adapter_4	AAGCGTAAGAATACGTGGCACAGACtttggatatcactcattagtggt
peaks_adapter_5	AATATTTTTGAATGGCTATTAGTCTtttggatatcactcattagtggt
peaks_adapter_6	TTAATGCGCGAACTGATAGCCCTAAtttggatatcactcattagtggt
peaks_adapter_7	AACATCGCCATTAAAAATACCGAACtttggatatcactcattagtggt
peaks_adapter_8	GAACCACCAGCAGAAGATAAAACAGtttggatatcactcattagtggt
peaks_adapter_9	AGGTGAGGCGGTCAGTATTAACACCtttggatatcactcattagtggt
peaks_adapter_10	GCCTGCAACAGTGCCACGCTGAGAGtttggatatcactcattagtggt
peaks_adapter_11	TCCCTTAGAATCCTTGAAAACATAGtttggatatcactcattagtggt
peaks_adapter_12	CGATAGCTTAGATTAAGACGCTGAGtttggatatcactcattagtggt
peaks_adapter_13	AAGAGTCAATAGTGAATTTATCAAAtttggatatcactcattagtggt
peaks_adapter_14	ATCATAGGTCTGAGAGACTACCTTTtttggatatcactcattagtggt
peaks_adapter_15	TTAACCTCCGGCTTAGGTTGGGTTAtttggatatcactcattagtggt
peaks_adapter_16	TATAACTATATGTAAATGCTGATGCtttggatatcactcattagtggt
peaks_adapter_17	AAATCCAATCGCAAGACAAAGAACGtttggatatcactcattagtggt
peaks_adapter_18	CGAGAAAACTTTTTCAAATATATTTtttggatatcactcattagtggt
peaks_adapter_19	TAGTTAATTTCATCTTCTGACCTAAtttggatatcactcattagtggt
peaks_adapter_20	ATTTAATGGTTTGAAATACCGACCGtttggatatcactcattagtggt
peaks_adapter_21	TACAGAGAGAATAACATAAAAACAGtttggatatcactcattagtggt
peaks_adapter_22	GGAAGCGCATTAGACGGGAGAATTAtttggatatcactcattagtggt
peaks_adapter_23	ACTGAACACCCTGAACAAAGTCAGAtttggatatcactcattagtggt
peaks_adapter_24	GGGTAATTGAGCGCTAATATCAGAGtttggatatcactcattagtggt
peaks_adapter_25	AGATAACCCACAAGAATTGAGTTAAtttggatatcactcattagtggt
peaks_adapter_26	GCCCAATAATAAGAGCAAGAAACAAtttggatatcactcattagtggt
peaks_adapter_27	TGAAATAGCAATAGCTATCTTACCGtttggatatcactcattagtggt
peaks_adapter_28	AAGCCCTTTTTAAGAAAAGTAAGCAtttggatatcactcattagtggt
peaks_adapter_29	GATAGCCGAACAAAGTTACCAGAAGtttggatatcactcattagtggt
peaks_adapter_30	GAAACCGAGGAAACGCAATAATAACtttggatatcactcattagtggt

Continued on next page...

Table S5: (continued)

ID	Sequence
peaks_adapter_31	AATAAGTTTTAACGGGGTCAGTGCCtttggatatcactcattagtggt
peaks_adapter_32	TTGAGTAACAGTGCCCGTATAAACAtttggatatcactcattagtggt
peaks_adapter_33	GTTAATGCCCCCTGCCTATTTCGGAtttggatatcactcattagtggt
peaks_adapter_34	ACCTATTATTCTGAAACATGAAAGTtttggatatcactcattagtggt
peaks_adapter_35	ATTAAGAGGCTGAGACTCCTCAAGAtttggatatcactcattagtggt
peaks_adapter_36	GAAGGATTAGGATTAGCGGGGTTTTtttggatatcactcattagtggt
peaks_adapter_37	GCTCAGTACCAGGCGGATAAGTGCCtttggatatcactcattagtggt
peaks_adapter_38	GTCGAGAGGGTTGATATAAGTATAGtttggatatcactcattagtggt
peaks_adapter_39	CCCGGAATAGGTGTATCACCGTACTtttggatatcactcattagtggt
peaks_adapter_40	CAGGAGGTTTAGTACCGCCACCCTCtttggatatcactcattagtggt
peaks_adapter_41	AAATCCGCGACCTGCTCCATGTTACtttggatatcactcattagtggt
peaks_adapter_42	TTAGCCGGAACGAGGCGCAGACGGTtttggatatcactcattagtggt
peaks_adapter_43	CAATCATAAGGGAACCGAACTGACCtttggatatcactcattagtggt
peaks_adapter_44	AACTTTGAAAGAGGACAGATGAACGtttggatatcactcattagtggt
peaks_adapter_45	GTGTACAGACCAGGCGCATAGGCTGtttggatatcactcattagtggt
peaks_adapter_46	GCTGACCTTCATCAAGAGTAATCTTtttggatatcactcattagtggt
peaks_adapter_47	GACAAGAACCGGATATTCATTACCCtttggatatcactcattagtggt
peaks_adapter_48	AAATCAACGTAACAAAGCTGCTCATtttggatatcactcattagtggt
peaks_adapter_49	TCAGTGAATAAGGCTTGCCCTGACGtttggatatcactcattagtggt
peaks_adapter_50	AGAAACACCAGAACGAGTAGTAAATtttggatatcactcattagtggt
peaks_adapter_51	GCGAACGAGTAGATTTAGTTTGACCtttggatatcactcattagtggt
peaks_adapter_52	ATTAGATACATTTCGCAAATGGTCAtttggatatcactcattagtggt
peaks_adapter_53	ATAACCTGTTTAGCTATATTTTCATtttggatatcactcattagtggt
peaks_adapter_54	TTGGGGCGCGAGCTGAAAAGGTGGCtttggatatcactcattagtggt
peaks_adapter_55	ATCAATTCTACTAATAGTAGTAGCAtttggatatcactcattagtggt
peaks_adapter_56	TTAACATCCAATAAATCATACAGGCtttggatatcactcattagtggt
peaks_adapter_57	AAGGCAAAGAATTAGCAAAATTAAGtttggatatcactcattagtggt
peaks_adapter_58	CAATAAAGCCTCAGAGCATAAAGCTtttggatatcactcattagtggt
peaks_adapter_59	AAATCGGTTGTACCAAAAACATTATtttggatatcactcattagtggt
peaks_adapter_60	GACCCTGTAATACTTTTGCGGGAGAtttggatatcactcattagtggt
